# Non-random spatial organization of telomeres varies during the cell cycle and requires LAP2 and BAF

**DOI:** 10.1016/j.isci.2024.109343

**Published:** 2024-02-28

**Authors:** Debora Keller, Sonia Stinus, David Umlauf, Edith Gourbeyre, Eric Biot, Nicolas Olivier, Pierre Mahou, Emmanuel Beaurepaire, Philippe Andrey, Laure Crabbe

**Affiliations:** 1 Molecular, Cellular and Developmental Biology Department (MCD), Centre de Biologie Intégrative (CBI), University of Toulouse, CNRS, UPS, 31062 Toulouse, France; 2 Laboratory for Optics and Biosciences, École polytechnique, CNRS, INSERM, IP Paris, 91128 Palaiseau, France; 3 Université Paris-Saclay, INRAE, AgroParisTech, Institut Jean-Pierre Bourgin (IJPB), 78000 Versailles, France

**Keywords:** Chromosome organization, Membrane architecture, Molecular interaction

## Abstract

Spatial genome organization within the nucleus influences major biological processes and is impacted by the configuration of linear chromosomes. Here, we applied 3D spatial statistics and modeling on high-resolution telomere and centromere 3D-structured illumination microscopy images in cancer cells. We found a multi-scale organization of telomeres that dynamically evolved from a mixed clustered-and-regular distribution in early G1 to a purely regular distribution as cells progressed through the cell cycle. In parallel, our analysis revealed two pools of peripheral and internal telomeres, the proportions of which were inverted during the cell cycle. We then conducted a targeted screen using MadID to identify the molecular pathways driving or maintaining telomere anchoring to the nuclear envelope observed in early G1. Lamina-associated polypeptide (LAP) proteins were found transiently localized to telomeres in anaphase, a stage where LAP2α initiates the reformation of the nuclear envelope, and impacted telomere redistribution in the next interphase together with their partner barrier-to-autointegration factor (BAF).

## Highlights

• Interdisciplinary study combining microscopy, spatial modeling, and a targeted screen
• Multi-scale spatial organization of telomeres evolves throughout the cell cycle
• A switch in the proportions of peripheral/internal pools of telomeres
• LAP2 proteins and BAF are actors in telomere tethering to the NE

## Graphical abstract

**Figure:**
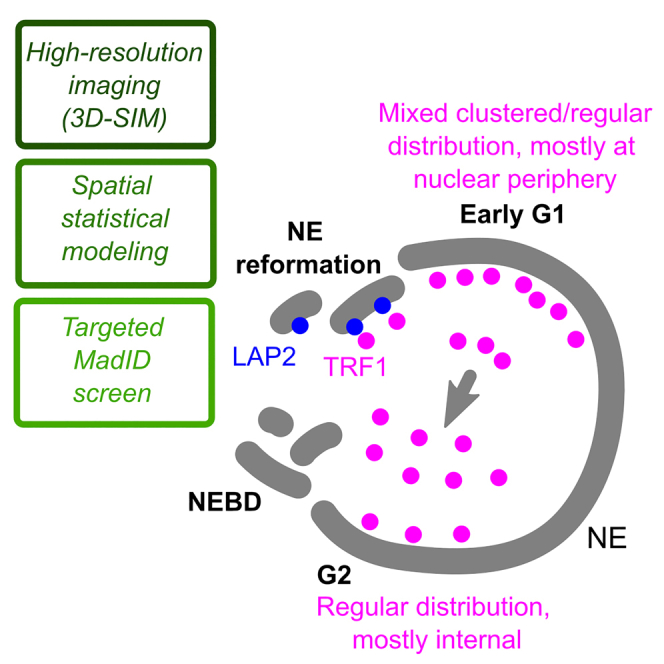

## Introduction

The organization of the genetic material within the nucleus influences major biological processes, ranging from the regulation of gene expression to the timing of DNA replication and the maintenance of genome stability. As such, spatial genome architecture can impact cell fate and must be transmitted through cell lineages in order to maintain cellular identity. The nuclear envelope (NE), which defines the boundaries of the nuclear volume, confers the essential scaffold required to organize the nuclear content. In metazoan cells, the inner side of the NE is lined with a meshwork of intermediate filaments polymers forming the nuclear lamina, composed of A- and B-type lamins.^1^ Together with additional NE-associated factors, the lamina provides a docking site for chromatin and creates a regulation hub for several essential cellular functions, including the establishment of the euchromatic active and heterochromatic inactive compartments within the nucleus, termed the A and B compartment, respectively.^2^^,^^3^ This organization in specific compartments is visible as more compacted heterochromatic regions close to the NE, and the “lighter” more decompacted euchromatic compartments toward the center of the nucleus. Within these compartments, many local and long-range contacts further organize chromatin in loops and topologically associated domains (TADs).

How 3D organization is established and what drives chromatin segregation by scaffolding are still unclear. Recent evidence suggests that centromeres, whose primary function is to promote proper chromosome segregation during cell division, could directly impact spatial genome architecture.^4^ Many studies in the budding yeast *Saccharomyces cerevisiae* and fission yeast *S. pombe* have clearly established a strong clustering of centromeres all along the cell cycle,^5^^,^^6^^,^^7^ and, more recently,^8^^,^^9^^,^^10^ feature that is conserved in metazoans.^11^^,^^12^^,^^13^ Centromere clustering impacts chromatin organization by creating a sub-compartment within the nucleus, as well as a barrier to intrachromosomal arm interactions. Importantly, a frequent localization of centromere clusters toward the nuclear periphery was also reported.^4^ In yeast, centromeres cluster in one focus near the spindle pole body, opposite the nucleolus, with a strong impact on genome spatial regulation. Studies in mice and humans also pointed to a localization of centromeres at the nuclear periphery. In human lymphocytes, 62% of pericentromeric heterochromatin was found in the B compartments associated with the lamina and the nucleolus.^13^

Similar to centromeres, the nuclear position of telomeres, ribonucleoprotein complexes located at the ends of linear chromosomes has also been intensely studied in yeast and has influenced the search for potential connections of mammalian telomeres with nuclear structures. In *S. cerevisiae*, telomeres are organized as clusters tethered to the inner nuclear membrane (INM) via the Sad1 and UNC84 (SUN)-domain protein Mps3,^14^ which promotes telomere silencing and the inhibition of unwanted recombination events between telomeric repeats.^15^^,^^16^ Telomere dynamics also play an essential function during meiosis in budding yeast, fission yeast, and mice, as their clustering to the NE is crucial for proper meiotic pairing and recombination of homologous chromosomes.^5^^,^^17^^,^^18^^,^^19^ By contrast, nuclear distribution of human telomeres is still very elusive. The dynamic behavior of human telomeres was previously studied over a period of a few hours, which uncovered their constrained diffusive movement, and the formation of dynamic clusters.^20^ 3D-fluorescence *in situ* hybridization (FISH) confocal microscopy experiments performed on human lymphocytes showed that telomeres are on average nearer to the center of the cell than centromeres and are not enriched at the nuclear periphery in interphase cells.^13^^,^^21^ A similar method followed by quantitative analysis to determine nuclear telomeric organization on fixed mouse and human lymphocytes established that telomeres assemble into a telomeric disk specifically in the G2 phase, suggesting a cell-cycle regulation aspect of telomere position.^22^ Cell-cycle regulation of telomere positioning was later evidenced by spinning-disk confocal time-lapse microscopy over an entire cell cycle. It was uncovered that ∼45% of human telomeres are physically attached to the NE during postmitotic nuclear assembly, in both primary fibroblasts and cancer cells.^23^ However, distance analyses were performed in 2D, using the middle plane of the cell, which corresponds only to a representative fraction of all telomeres within the 3D nuclear volume.

Mitosis represents one of the greatest challenges to maintain cellular identity. While chromatin is condensed up to 50-fold in metaphase chromosomes, TAD and chromosome compartments are lost at this stage.^24^ In addition, NE breakdown that characterizes open mitosis totally resets nuclear structure. During postmitotic nuclear assembly, nuclear size needs to be readjusted, nuclear pores that allow trafficking between the nucleus and the cytoplasm are reinserted within the NE, and chromosome territories are established.^25^ These events must be finely coordinated to ensure that segregated DNA is finally enclosed in a single-cell nucleus in each daughter cell. During late anaphase/telophase, INM proteins bind chromatin to initiate attachment of membrane sheets.^26^ Early live microscopy studies using fluorescently tagged proteins expressed in HeLa cells found that chromosome ends associate transiently to the INM proteins lamina-associated polypeptide (LAP) 2α isoform and barrier-to-autointegration factor (BAF).^27^ These results are in accordance with the observation that telomeres decorate the NE in early G1, where they are found transiently interacting with lamins, LAP2α, and emerin specifically during postmitotic nuclear reformation.^23^

Here, we propose an approach to perform a systematic analysis of telomere spatial positioning within the nucleus and across the cell cycle to shed light on the role of telomeres in nuclear organization. We used a pipeline combining high-resolution 3D imaging of telomeres and NE proteins at specific cell-cycle stages, complemented with 3D spatial statistics and modeling.^28^^,^^29^ We found that telomeres are organized in a non-random fashion in the nucleus and are undergoing a dynamic repositioning from the periphery to the interior as cells progress from early G1, through G1/S and G2. By contrast centromeres remained predominantly associated with the periphery in a polarized way. We then conducted a targeted screen using MadID, a proximity labeling approach to map protein-DNA interaction,^30^^,^^31^ that revealed a set of factors involved in telomere tethering to the NE. Among these factors, we further showed that under endogenous conditions LAP2 proteins associated with telomeres in anaphase, at the onset of NE reformation. Co-depletion of BAF and LAP proteins affected the size of reforming nuclei after mitosis, and the nuclear distribution of telomeres in the subsequent interphase.

## Results

### 3D structured illumination microscopy (3D-SIM) to assess telomere and centromere spatial organization

**Figure 1:**
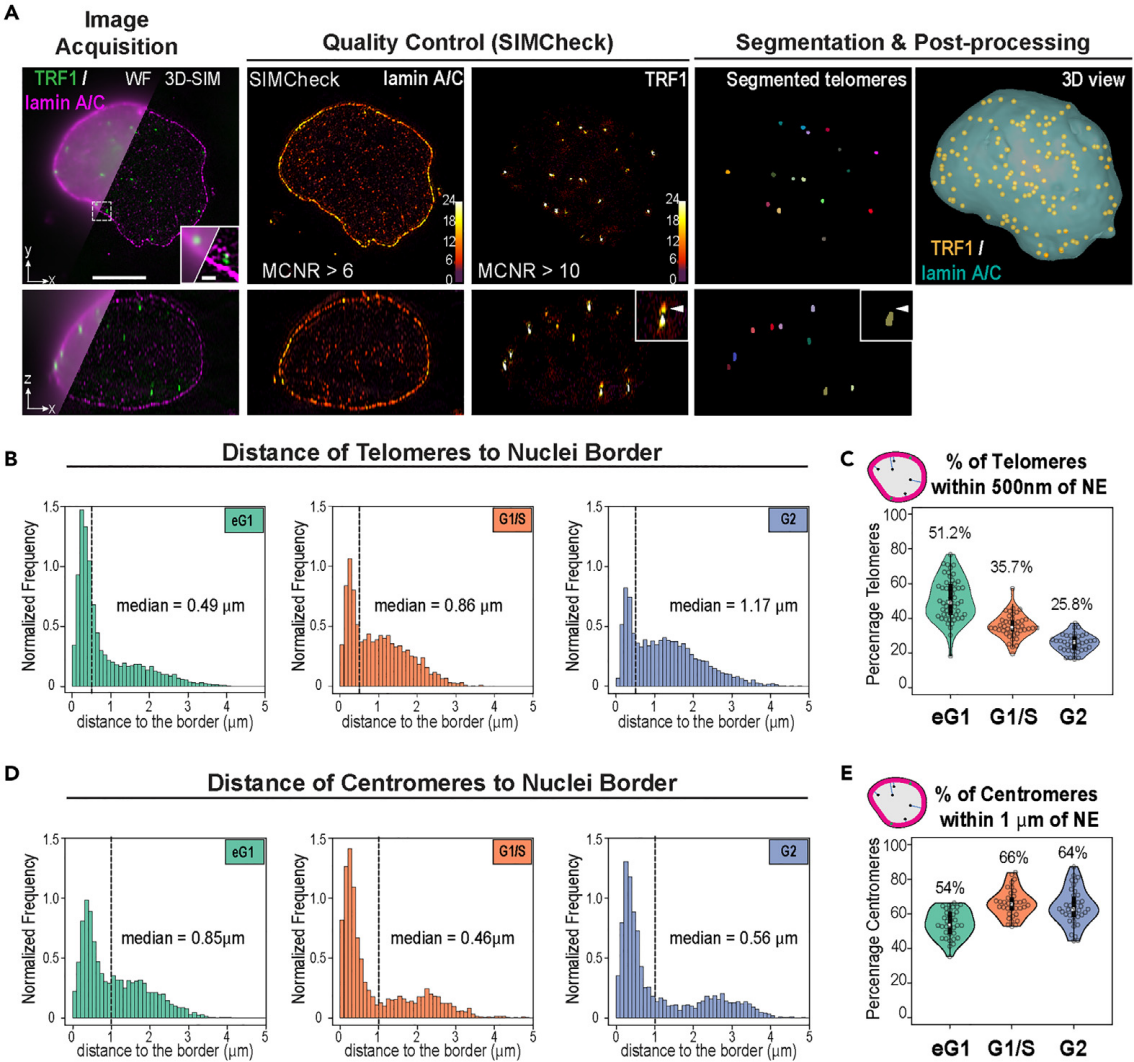
Quantitative analysis of 3D-SIM images of HeLa cell nuclei shows dynamic positioning of telomeres during cell cycle (A) Overview of the analysis pipeline from image acquisition to segmentation and post-processing. Representative images of nuclei stained for TRF1 (*green*) and lamin A/C (*magenta*) highlighting the increased resolution in 3D-SIM compared to pseudo widefield (WF, top left) in the lateral *xy* and axial *xz* directions. The quality of 3D-SIM images was assessed using SIMCheck; cells with adequate modulation contrast (MCNR) values for lamin A/C and TRF1 were then segmented and processed. Telomeres segmented with a specifically developed pipeline described in the STAR Methods are shown in individual XY and XZ sections. *Inset*: artifactual echoes in the SIM reconstruction are removed by the segmentation pipeline. 3D view shows segmented telomeres (*Yellow dots*) within a segmented nucleus (*Blue surface*). Scale bars: 5 μm (main) and 1 μm (inset). (B) Distribution of distances between telomeres and nuclear border during cell cycle and their median value showing increased distance to the NE (early G1 phase, N = 54 nuclei; G1/S phase, N = 43 nuclei; G2 phase, N = 39 nuclei). Distance was measured from the center of each telomere to the closest point at the nuclear surface. Frequencies were normalized to obtain a unit area histogram. The dotted line is set at 0.5 μm and represents the estimated thickness of the nuclear lamina but also correspond to the cutoff between the two pools of telomeres. (C) Distribution of percentage of telomeres located at a distance below 500 nm from nuclear border in individual nuclei during cell cycle. (D) Same as B for centromeres (N = 35, 36, and 38 for early G1, G1/S, and G2 phases, respectively). The dotted line is set at 1 μm and corresponds to the cutoff between the two pools of centromeres. (E) Distribution of percentage of centromeres located at a distance below 1 μm from nuclear border in individual nuclei during cell cycle.

To study the organization of telomeres and centromeres within the nucleus at given cell-cycle stages and with high 3D resolution, we turned to 3D-SIM, as it allows rapid multi-color imaging over the depth of a cell at 8-fold increased volumetric resolution over the diffraction limit.^32^ To obtain a high signal-to-noise ratio for telomere segmentation, we used HeLa cells expressing TRF1, one of the core proteins from the Shelterin complex that sits on telomeric repeats, tagged with EGFP. HeLa cells are widely used in the field, and telomere capping and cell-cycle progression were previously shown to be unaffected in EGFP-TRF1-overexpressing HeLa cells.^23^ Centromeres were stained using autoantibodies specific to centromeres found in CREST syndrome (CREST), and the NE was visualized by co-staining of lamin A/C, a component of the nuclear lamina, and SUN1, a transmembrane protein localized at the INM. Following synchronization, cells were fixed for immunofluorescence at the G1/S boundary, in G2 phase, or 8.5 h after release from the G1/S block to enrich in cells in late mitosis/early G1 phase (Figures S1A and S1B). Early G1 cells that are still in the process of postmitotic nuclear assembly displayed the typical SUN1 aggregates around the nucleus, corresponding to the portion of the NE protein not yet localized at the envelope (Figure S1B).^23^ Early G1 cells readily reach sizes of about 15–20 μm, thus often leading to decreased 3D-SIM image contrast. To circumvent this effect, we devised a 3D-SIM mounting medium based on the previously reported clearing agent sorbitol^33^ and assessed 3D-SIM modulation contrast by SIMCheck^34^ and reconstruction quality (Figure 1A) prior to analysis. We quantitatively assessed in 3D various morphometric descriptors based on a specifically devised image processing and segmentation pipeline, including an echo-suppression algorithm (Figure 1A and STAR Methods). Segmentation was performed on images of individual nuclei from two datasets stained for nuclear lamina and either telomeres or centromeres.

For both datasets, we confirmed that the nuclear morphology varied across the cell cycle (Figures S1D and S1E). The volume of late mitosis and early G1 nuclei exhibited a bimodal distribution, corresponding to the transition from telophase (<∼800 *μ*m^3^) to a more decompacted state where nuclei are bigger (>∼800 μm^3^). Nuclear volume almost doubled when transitioning from early G1 to G2 (from 800 μm^3^ G1/S nuclei to 1,300 μm^3^ G2 nuclei). Centromere size and shape also varied during the cell cycle, with volumes ranging from 0.21 ± 0.01 μm^3^ in early G1 to 0.27 ± 0.01 μm^3^ in G2 (Figure S1E). By contrast, the number of segmented centromeres decreased from 84 ± 2 in early G1 to 67 ± 3 in G1/S and ∼67 ± 1 in G2. The concomitant decrease of centromere number and increase of their volume point to centromere clustering that cannot be resolved in 3D-SIM, also observed in previous studies,^11^^,^^13^ or reflect the structural evolution of the centromeric complex as described for Centromere Protein A (CENP-A), transitioning from a globular rosette in eG1 to a less structured and wider disc in early mitosis.^35^ Telomere number also varied throughout the cell cycle (Figure S1D). We segmented 142 ± 2 telomeres per nucleus in early G1, which correspond to the ∼70 chromosomes described in HeLa cells. The number of telomeres decreased to 65 ± 2 and 91 ± 3 in G1/S and G2 phases, respectively. This suggests that telomeres also undergo clustering, as previously observed.^22^^,^^23^^,^^36^^,^^37^ Concomitant to the increase in nuclear volume, this trend resulted in an overall decrease of telomere density between early G1 and G2. Overall, our results confirmed that 3D-SIM combined with specific image analysis pipelines was a valid approach to quantify in 3D and with a high resolution the dynamics of telomeres and centromeres during the cell cycle.

### Spatial statistical modeling shows dynamic positioning of telomeres relative to the NE over the cell cycle while centromeres remain stably associated

To study telomere distribution, we first analyzed the distance of each telomere to the edge of the nucleus in early G1, G1/S, and G2 cells (Figure 1B). More than 50% of telomeres were found within 500 nm of the NE in early G1, with a median distance to the NE of 0.49 μm (Figures 1B and 1C), in accordance with 2D image analysis.^23^ In addition, our analysis further showed that telomere distance to the NE exhibited a bimodal distribution in all three cell cycle phases, revealing a pool of telomeres localized close to the NE and a pool of more internally located telomeres with a larger spread of distances. In S and G2, the cutoff between these two pools was at ∼500 nm from the NE (dotted line), the estimated thickness of the lamina-associated domains (LADs) contacting the nuclear lamina.^38^ In addition, the proportion of detected peripheral telomeres gradually decreased during the cell cycle, to reach ∼36% and ∼26% of telomeres within 500 nm of the NE in G1/S and G2, respectively. These results suggest the existence of two sub-populations of telomeres that undergo a dynamic switch between the periphery and the nuclear interior as cells progress through the cell cycle, with only ∼20 detected telomeres that remained near the NE (Figure S1D). Centromeres also exhibited a bimodal distribution of distance to the nuclear border, with a cutoff between peripheral and internal centromeric sub-populations at ∼1 μm of the border (Figure 1D, dotted line), likely due to the 10-fold larger size of centromeres compared to telomeres and accompanying steric constraints. However, unlike telomeres, the proportion of peripheral centromeres increased from 54% in early G1 to 66% in G1/S and remained stable at 64% in G2 (Figure 1E). Absolute sub-population sizes showed this trend was due to a decrease in the number of detected internal centromeres at G1/S, while the number of peripheral centromeres remained stable throughout the cell cycle (Figure S1E), indicating a more stable proximity to the NE.

**Figure 2:**
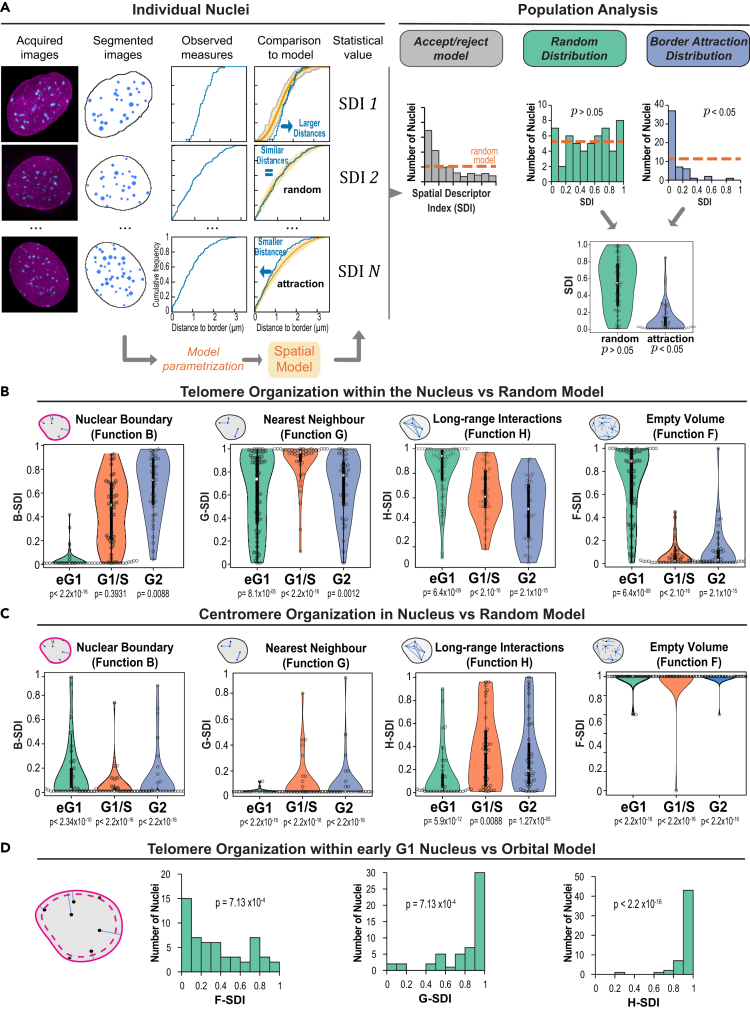
Statistical spatial analysis shows a dynamic switch in the spatial interactions between telomeres and nuclear envelope during the cell cycle (A) Statistical spatial analysis pipeline: illustration with the testing of spatial interaction with the nuclear border. Based on the cumulative distribution function (CDF) of the distance between telomeres and nuclear border, observed individual patterns were compared to predicted patterns under a random model of organization. For each pattern, the probability of observing smaller distances under the model than actually observed was computed (spatial descriptor index, SDI). Upon a positive spatial interaction (attraction) between telomeres and nuclear border, small values of the SDI are expected because smaller distances should be observed as compared to model predictions. In the absence of any spatial interaction, a uniform distribution is expected (*Orange dotted line*). The Kolmogorov-Smirnov goodness-of-fit test is used to assess the uniformity of the population distribution of the SDI. (B) Analysis of spatial interactions between telomeres and nuclear border or between telomeres using comparisons to the random model of telomere organization. *Function B*: distribution of SDI computed using the CDF of the distance between each telomere and nuclear border. *Function G*: distribution of SDIs computed based on the cumulative distribution function of the distance between each telomere and its closest neighbor. Function H: distribution of SDIs computed based on the distance between each telomere and any other telomere. Function F: distribution of SDIs computed based on the distance between arbitrary nuclear positions and their closest telomeres. (p: p value of Kolmogorov-Smironov test of uniformity). Early G1 phase, N = 54 nuclei; G1/S phase, N = 43 nuclei; G2 phase, N = 39 nuclei. (C) Same as B for centromeres. Early G1 phase, N = 35 nuclei; G1/S phase, N = 36 nuclei; G2 phase, N = 38 nuclei. (D) Analysis of spatial interactions between telomeres in early G1 using comparisons to the orbital model of telomere organization (N = 54 nuclei). The scheme illustrates the orbital model, which is similar to the completely random model of telomere organization except that the observed distance between each telomere and nuclear border is preserved.

The radial positioning of chromatin is functionally relevant, as the nuclear periphery is known to be a domain boundary that regulates chromatin function and organization. However, though they provide valuable information, distance measurements are not sufficient to assess spatial proximity with nuclear border. For example, about 90% of the volume in a sphere is closer to the border than to the center.^28^ Therefore, we adopted a statistical spatial modeling approach recently developed to assess 3D spatial interactions in object patterns.^28^ Here, spatial interactions mean interdependent positioning between objects or between objects and the NE (for example attraction or repulsion), which does not necessarily imply direct physical contact. In this method, the observed 3D spatial distribution of objects within a single nucleus is quantitatively described using distance functions (detailed in the following) and compared to a theoretical distribution model of those same objects within this same nucleus (Figure 2A). The difference between the observed pattern and model predictions is quantified by a spatial distribution index (SDI) that varies between 0 (observed distances far below predictions) and 1 (observed distances far above predictions). At the population level, the model is rejected if the SDI distribution differs from the uniform distribution between 0 and 1 (Figure 2A). We first considered the distance of objects to the nuclear periphery using the B function (Figure S2A). Using this function to compare observed patterns to patterns predicted under the completely random model, in which positions are uniformly and independently distributed within the nuclear space (see STAR Methods), allowed us to test the null hypothesis that telomere positions were independent from the NE. We found that in early G1 nuclei the positioning of telomeres relative to the periphery strongly deviated from the random model, with smaller distances to the nuclear boundary than expected under randomness (Figure S2B). This positive spatial interaction with NE in early G1 was confirmed at the population level with a skewed distribution of B-SDI values toward 0 (Figure 2B). Preferential association of telomeres with the periphery was lost once cells were at the G1/S boundary (Figure 2B). Analysis of G2 nuclei showed an inverted pattern of interactions with NE compared with early G1 nuclei, with telomeres exhibiting larger distances from the NE than under the random model. The C function, which probes the distance of objects to the center of nuclei (Figure S2A), corroborated these results and the switch from globally positive to globally negative spatial interactions between telomeres and NE between early G1 and G2 (Figure S2C). Taken together, our analysis revealed a switch in the radial positioning of telomeres and in telomere-NE spatial interactions during the cell cycle, with first a decrease in the number of peripheral telomeres followed by an increase in the number of internal telomeres as cells progress from early G1 to G2. Centromeres behaved very differently and exhibited a consistently strong and positive non-random spatial interaction with the nuclear periphery that was maintained throughout the cell cycle and even reinforced from G1/S with the decrease in the number of internally located centromeres (Figures 2C, S2D, and S2E).

### Polarity in telomere and centromere distribution

**Figure 3:**
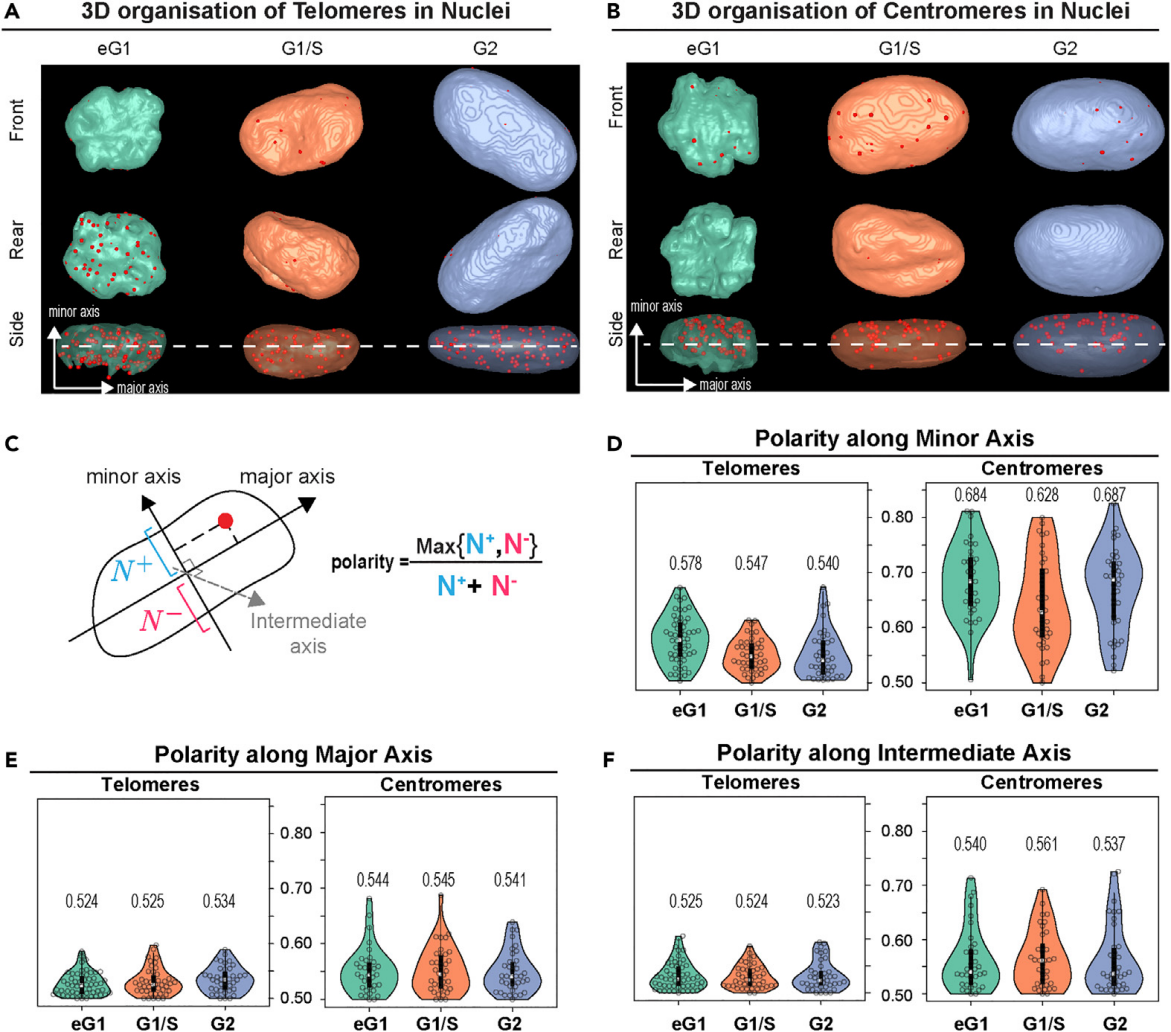
Polarity analysis of telomere and centromere nuclear organization across the cell cycle (A) 3D views of segmented sample nuclei (*Color surfaces*) and their telomeres (*Red spots*) at different phases of the cell cycle. Nuclear surfaces are displayed opaque in front and rear views and are transparent in side views. The minor axis is orthogonal to the plane of view in front and rear views. The dotted line represents the middle plane of the cell that would be used on a 2D based analysis. (B) Same as A for centromeres. (C) Schematic of computation of the polarity index along the minor axis of the nucleus. Positions are projected along a given axis, and the proportions of projections located above and below the center of the nucleus are computed. The polarity index is the largest of these two proportions. It can be computed for the major, minor, or intermediate axes. (D) Cell-cycle distribution of the polarity index along the minor nuclear axis for telomeres and centromeres. Median values are indicated. The Kruskal-Wallis rank-sum test showed significant effect only for telomere polarity along the minor axis (p = 0.0006). *Post hoc* comparison tests (Wilcoxon test with Benjamini-Hochberg correction for multiple testing) showed significant difference between early G1 and the two other stages (eG1 vs. G1/S: p = 0.002; eG1 vs. S/G2: p = 0.002; G1/S vs. S/G2: p = 0.534). For telomeres N = 54, 43, and 39 for early G1, G1/S, and G2 phases, respectively. For centromeres N = 35, 36, and 38 for early G1, G1/S, and G2 phases, respectively. (E) Same as D for polarity index along the major axis. (F) Same as D for polarity index along the intermediate axis.

We took a closer look at the 3D organization of both telomeres and centromeres and observed a difference in polarity along the minor axis of the nucleus. Early G1 telomeres were predominantly found on one side of the nucleus while centromeres were located on the opposite side (Figures 3A and 3B), which is likely a consequence of the pulling forces exerted on centromeres during anaphase that lead to a Rabl-like configuration. We therefore quantitatively assessed the polarity of telomeres and of centromeres along each of the three main axes of the nucleus and defined a polarity index based on the proportion of positions that projected on each half-axis (Figure 3C). By construction, the polarity index for a given axis takes its minimum value 0.5 if the positions are equally distributed between the two axis halves and takes its maximum value 1 if all positions are concentrated on one-half. The polarity analysis confirmed the early G1 asymmetric distribution of both telomeres and even more so of centromeres along the minor axis of the nucleus and the absence of polarity along the two other axes (Figures 3D–3F). However, while telomeres lost their polar organization in later stages, centromeres exhibited a sustained high polarity index. These data suggest that a Rabl-like configuration of centromeres is, to some extent, preserved during the whole cell cycle. This observation could not be made based on the 2D analysis of the middle plane of the cell (Figures 3A and 3B, dotted line) that was previously performed.^23^ Overall, our results stress the importance of coupling 3D high-resolution imaging and quantitative image analysis to unbiased statistical schemes that assess spatial interactions at the population level despite heterogeneity in nuclear morphology and in the number and sizes of nuclear objects.

### Telomeres exhibit a multi-scale organization in the nuclear volume in early G1

We next asked whether additional spatial interactions were characterizing telomere organization. We relied on the same spatial modeling approach as earlier, comparing observed patterns to predictions of the random distribution model using distance functions that probe the relative positioning between objects. We first analyzed the distance between each object and its nearest neighbor, using the function G (Figure S2A). In early G1, telomeres showed a mixed behavior with an attractive trend in the short-distance range (shorter distances to nearest neighbor than under the random model at distances < ∼1 μm) and a repulsive trend, characteristic of a regular distribution, in the long-distance range (larger distances than under the model at distances >1 μm) (Figures S3A and S3B). This mixed response (attraction and repulsion) was reflected at the population level by the bimodal distribution of the corresponding G-SDI (Figure 2B). The long-range regularity was confirmed by analyzing the distances between all pairs of telomeres (function H; Figure S2A), which showed on average larger distances than expected under a random organization, both on individual patterns (H-H0>0 at distances >2 μm – Figure S3C) and at the population scale (Figure 2B). Function F, which probes the empty spaces between objects (Figure S2A), showed that at both the individual and the population levels the voids between telomeres were larger than expected under randomness (Figures S3D and 2B), thus confirming the trend for clustering in the short-range scale. Overall, the three different spatial descriptors suggest a multi-scale organization of telomeres in early G1, with telomeres being separated by large regions in the nuclear space yet showing a regular, repulsive-like distribution in the domains they occupy.

### Telomere organization is subtended by mutually repulsive spatial interactions

We wondered whether the preferentially peripheral positioning of telomeres in early G1 was sufficient to explain the short-range attraction or, conversely, the long-range repulsion. We evaluated these hypotheses by comparing observed patterns to predicted distributions under the orbital model.^28^ This model enforces identical relative positioning to the nuclear border in both observed and predicted patterns. The SDI distributions obtained with the three distance functions all pointed to a more regular distribution of telomeres than predicted under a purely orbital distribution and showed no tendency for clustering (Figure 2D). This shows that, in the comparison to the aforementioned completely random model, the demonstrated attractive effect was probably due to the clustering of telomeres at the nuclear periphery while the repulsive effect could not. Hence, the orbital model confirms that telomere organization is not only radial but also characterized by mutually repulsive spatial interactions.

As cells progressed through the cell cycle, the mixed behavior observed in early G1 with both attraction and repulsion was lost. In G1/S, observed distances to nearest neighbor were larger than expected under the random model (Figures S3B and 2B). The loss of the attractive trend was confirmed by analysis using functions F and H, which showed more regular and smaller voids between telomeres and larger inter-distances between telomeres, respectively, than expected under a random model, thus pointing to a regular distribution (Figures S3C, S3D, and 2B). Functions F and G showed that regularity was maintained in G2 at least in the short-distance range, since the observed H-SDI distribution did not differ from complete randomness (Figure 2B). Thus, at the G1/S transition and G2, telomeres exhibited a purely repulsive and regular organization, devoid of clustering. Overall, telomeres followed throughout the cell cycle a more regular distribution than expected under a random organization, a possible consequence of the partition of the nucleus into chromosome territories. In early G1, a clustering effect superimposed onto this repulsive pattern, likely due to the peripheral polar organization of telomeres along the nucleus minor axis.

From early G1 to G2, distances between each centromere and its nearest neighbor were consistently smaller than expected under a random model (Figure 2C), thus showing a preserved trend for a clustered organization. This attractive pattern was confirmed by the larger spaces devoid of centromeres evidenced by function F and by the globally smaller inter-distances between centromeres shown by function H (Figure 2C). Taken together, our spatial analyses confirm a strong clustering of centromeres in addition to their predominantly peripheral localization within the nucleus at all cell-cycle stages and show a more stable organization throughout the cell cycle than observed for telomeres.

### MadID-based targeted screen reveals factors involved in telomere-NE anchoring

**Figure 4:**
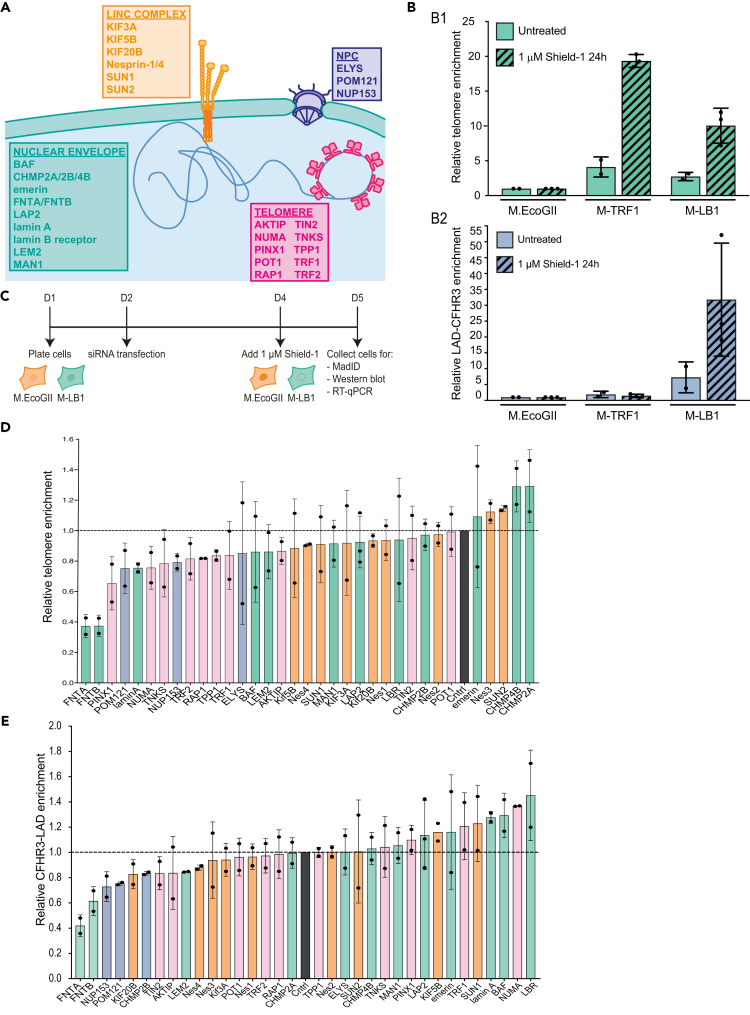
MadID-based targeted screen reveals factors involved in telomere interaction with the nuclear envelope (A) Scheme of proteins selected for the targeted MadID screen. Selected proteins belong to four categories: members of the linker of nucleoskeleton and cytoskeleton complex (LINC, orange), members of the nuclear pore complex (blue), members of the shelterin complex (magenta), and nuclear envelope-related proteins (green). (B) Relative telomere (top) and LAD-CFHR3 (bottom) enrichment in cells expressing M.EcoGII, M-TRF1, or M-LB1, in absence (n = 2) or presence (n = 3) of Shield-1 (1 μM, 24h). Mean ± SD is shown. (C) Experimental setup of MadID-based targeted screen. (D) Relative telomere enrichment upon depletion of the indicated proteins. The mean telomere enrichment is shown relative to iLAD-SMIM2 and to control condition (set at 1). The whiskers represent the standard deviation. Individual values are shown as black circles. n = 2 except for LAP2 (n = 3). For simplification, Nesprin-1/4 were labeled as Nes 1–4. (E) Relative LAD-CFHR3 enrichment upon depletion of the indicated proteins. The mean LAD-CFHR3 enrichment is shown relative to iLAD-SMIM2 and to control condition (set at 1). The whiskers represent the standard deviation. Individual values are shown as black circles. n = 2 except for LAP2 (n = 3).

Next, we aimed to identify the actors involved in 3D telomere organization and in telomere tethering to the NE. We took advantage of MadID, a tool we recently developed to probe for protein-DNA interactions *in vivo* using proximity labeling.^30^^,^^31^ MadID relies on the expression of the bacterial methyltransferase M.EcoGII, which adds methyl groups to N6-adenosine (m6A) in any DNA sequence context. When fused to lamin B1, M.EcoGII specifically methylates chromatin that comes in contact to the nuclear lamina. This technique was previously used to map LADs by whole-genome sequencing with high specificity, and deep and unbiased genome coverage.^30^ MadID could also probe telomeres-NE contact sites in human cells, which we decided to use as readout for a small interfering RNA (siRNA)-mediated targeted screen. We selected 34 targets, either because of their suspected role in chromatin organization or after a mass spectrometry screen we performed to uncover hits involved in telomere-NE anchoring (unpublished work). These factors could be classified in four different groups: i) members of the shelterin complex and related proteins, ii) members of the linker of nucleoskeleton and cytoskeleton (LINC) complex, iii) members of the nuclear pore complex (NPC), and iv) proteins from the NE and related (Figure 4A). HeLa cells were transduced with inducible retroviral vectors expressing either M.EcoGII-lamin B1 (M-LB1) or untargeted M.EcoGII that was used as a reference to correct for local differences in chromatin accessibility. Clonal cell populations expressing equal levels of M.EcoGII or M-LB1 were isolated. Following 24 h of Shield-1-dependent induction, M-LB1 was found properly localized at the nuclear rim (Figure S4A) and catalyzed genomic DNA methylation detected with an m6A-specific antibody *in situ* by DNA-immunofluorescence (DNA-IF) (Figure S4B) and immuno-dot-blot (Figure S4C).^30^^,^^31^ To test whether MadID could be performed in these clonal cells with high specificity and reproducibility, we performed m6A-specific immunoprecipitations (m6A-IPs) followed by quantitative PCR (qPCR) using primers specific to the well-established LAD-CFHR3 and inter-LAD-SMIM2 regions (iLAD),^39^ as well as to telomeric repeats.^40^ We found a 30-fold enrichment of LAD-CFHR3 and a 10-fold enrichment of telomeric repeats specifically in M-LB1 cells, further confirming the proximity of a subset of telomeres to the NE (Figure 4B). By contrast, HeLa cells expressing M.EcoGII fused to the shelterin protein TRF1 (M-TRF1) to directly address the methyltransferase to telomeres,^30^ triggered methylation of telomeric repeats but not of the LAD-CFHR3 region (Figure 4B).

After validation of the clonal cell populations, we implemented the screen following the experimental setup as shown in Figure 4C. Briefly, clonal cells were transfected with siRNA for 72 h, and M.EcoGII or M-LB1 expression was induced by addition of Shield-1 during the last 24 h before collection. Efficiency of target depletion could be verified for 32/34 the candidates by western blotting or RT-qPCR, with the exception of POM121 and TIN2 for which no specific antibodies or good PCR primers were found (Figures S4H and S4I). m6A-IP followed by qPCR was then performed to reveal telomeric and LAD enrichments in the IP fraction. Importantly, cycle threshold values for all three regions as well as the relative fold enrichments over SMIM2 obtained for siControl replicates were highly reproducible across multiple independent siRNA experiments (Figures S4D and S4E). We then plotted the relative telomere and LAD-CFHR3 enrichments for all tested siRNA relative to siControls (Figures 4D and 4E). The obtained graph indicates whether the depletion of each target decreased (values below 1) or increased (values above 1) contact frequencies between LAD-CFHR3 or telomeric repeats and lamin B1. To further assess the specificity of our screen, siRNA against farnesyltransferase (FNT) alpha and beta was used to perturb lamin B1 localization at the NE. A- and B-type lamins are major farnesylated proteins, and, while the mature form of lamin A loses its farnesylated site by cleavage during its final processing, lamin B1 and lamin B2 keep their farnesyl moiety. We therefore postulated that siRNA against FNTA or FNTΒ could prevent proper addressing of M-LB1 to the NE, similarly to the addition of farnesyl transferase inhibitors that mislocalize B-type lamins to the nucleoplasm.^41^ Indeed, we found a strong defect in M-LB1 addressing to the NE in cells depleted for either FNT (Figures S4F and S4G), and a 40% to 60% reduction in LAD-CFHR3 and telomeres enrichment in the m6A-IP fraction (Figures 4D and 4E).

Overall, we found that most selected targets had a positive effect on telomere tethering, as their depletion reduced telomere enrichment in the m6A-IP fraction compared to siControls (Figure 4D). Hence, depletion of members of the shelterin complex decreased contacts with the NE, suggesting that telomeric proteins engage, to some extent, in protein-protein interactions leading to telomere tethering. Except for TIN2, depletion of members of shelterin did not affect LAD-CFHR3 contacts (Figure 4E). Depletion of members of the LINC complex, such as SUN1, kinesins, and nesprins, also altered telomeres-NE contact sites. This result is coherent with the role of the LINC complex in telomere tethering in several organisms, as well as its role in chromatin movement during meiosis.^42^ A similar trend was found for the members of the NPC complex POM121, ELYS, and NUP153, which were previously shown to be involved in the regulation of genome architecture or NE reformation.^43^^,^^44^^,^^45^^,^^46^^,^^47^^,^^48^ In contrast, members of the ESCRT-III complex CHMP4B and CHMP2A seemed to prevent telomere anchoring, as their depletion increased telomere-lamin B1 interaction. Described for its role in cytoplasmic membrane fusion, the ESCRT-III complex is also known to remodel the NE, particularly during mitotic exit.^49^^,^^50^ CHMP2A is recruited to the reforming NE in late anaphase through CHMP4B, providing an essential function for NE reformation. Importantly, CHMP2B, which did not trigger a change in telomere methylation status, has minimal effect on NE reformation by itself and requires co-depletion with CHMP2A to trigger a greater phenotype.^49^ Among other proteins associated with the NE, lamin A and BAF or LAP proteins had an interesting effect on chromatin attachment to the NE. Indeed, their depletion increased the interaction between LAD-CFHR3 and lamin B1, as previously shown,^51^ but decreased telomere attachment (Figures 4D and 4E). Altogether, our screen identified new factors engaged in telomere organization via their tethering to the NE.

### LAP2α and LAP2β are recruited to telomeres in early anaphase

**Figure 5:**
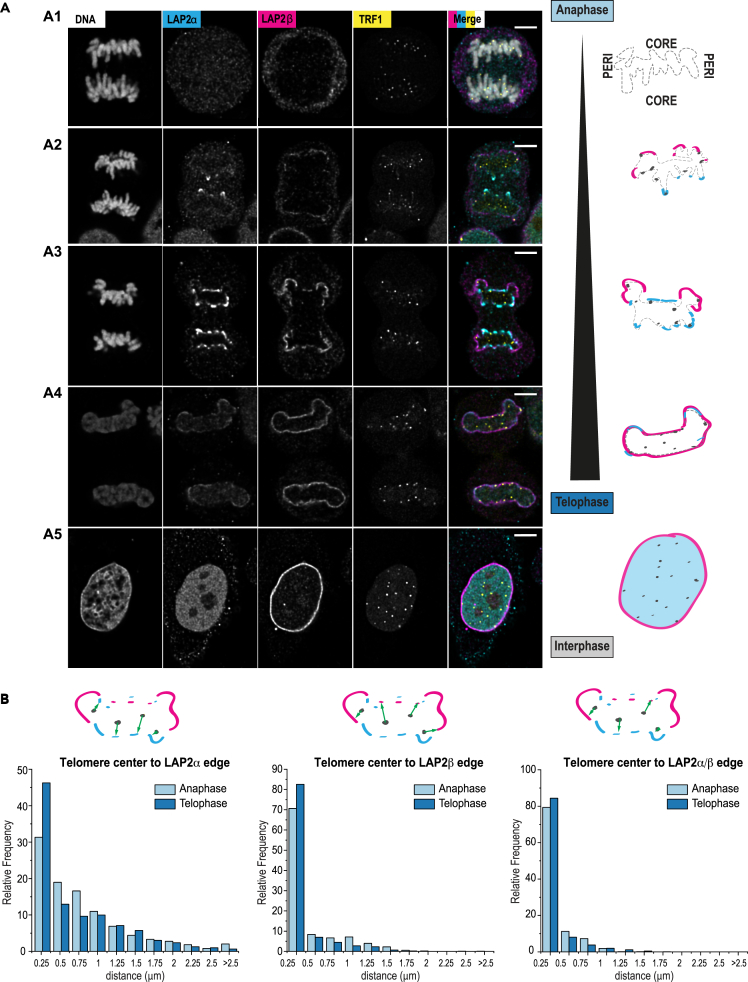
LAP2α and LAP2β are recruited to telomeres in early anaphase (A) Representative images and corresponding schematics illustrating the stepwise recruitment of LAP2α (*cyan*) and LAP2β (*magenta*) at telomeres (*TRF*1 – *yellow)* and chromosomes (DNA – *gray*) from anaphase to interphase. After being first un-detectable at anaphase onset (panel A1), LAP2α enriches at chromosome ends at telomeres and LAP2β is weakly detected at peripheral DNA (panel A2) followed by stronger enrichment of LAP2α at the core and LAP2β extends at the periphery (panel A3) until in Telophase all the DNA is encapsulated by LAP2β and LAP2α decreases at the nuclear envelope (panel A4). In interphase, LAP2β remains enriched at the nuclear envelope and LAP2α appears primarily nucleoplasmic (panel A5). Scale bars: 5 μm (main). (B) Histograms of relative frequencies (%) of telomeres’ distances from the center of mass of the segmented signal to the edges of segmented signal from LAP2α, LAP2β, or a segmentation mask combining both stainings (LAP2α + LAP2β). Segmentations were computed from deconvolved confocal images of cells in anaphase (typically with morphologies as in A3) and in telophase (morphologies as in A4). Error bar indicates standard error of the mean; N = 8 cells in anaphase, N = 12 cells in telophase; number of segmented telomeres per cells vary and are not indicated.

While our targeted screen provided a first hint on the molecular pathways driving or maintaining telomere-NE attachment in human cells, we sought to address the establishment of such interaction, which takes place at the end of mitosis. In late anaphase, membranes need to reform around chromatin, and evidence suggests that endoplasmic reticulum membrane tubules are targeted to chromatin through chromatin-binding NE proteins.^52^^,^^53^ The major NE protein association sites on chromatin, called peripheral or core regions, attract different sets of proteins by poorly understood mechanisms. A pioneer study using overexpression of fluorescently tagged proteins showed that LAP2α transiently localizes in anaphase to telomeres at inner core regions to initiate NE reformation.^27^ A subfraction of BAF is also relocalized to core structures together with LAP2α. These results suggest that telomere-NE anchoring observed during postmitotic nuclear assembly^23^ could initiate in anaphase, and that telomeres could serve as a nucleation point for membrane reformation. To test this hypothesis, we labeled endogenous LAP2α by immunofluorescence during all stages of mitosis and acquired images using confocal microscopy followed by deconvolution (Figure 5A). As expected, endogenous LAP2α was found mainly diffused in the nucleoplasm during interphase, and transiently recruited at core regions of chromatin early in anaphase, with accumulation of the protein at discrete sites that often overlapped with telomeric signal (Figures 5A and S5A). Instead, LAP2β isoform was rather observed at peripheral regions before the signal extends all around chromatin in later mitotic stages (Figures 5A and S5A). Following image segmentation, we quantified the distance between the centroid of telomeres and the edge of LAP2α/β-labeled regions using BIP software (see STAR Methods). Both LAP isoforms were positioned in close proximity to telomere foci in anaphase and telophase. About 79% of telomere foci in anaphase and 84% in telophase were found within 250 nm of LAP2 structures (Figures 5B and S5A). Altogether, these results suggest that NE proteins involved in membrane reformation are recruited at or close to telomeres during postmitotic nuclear assembly.

### Depletion of BAF and LAP2 proteins affects 3D genome organization

**Figure 6:**
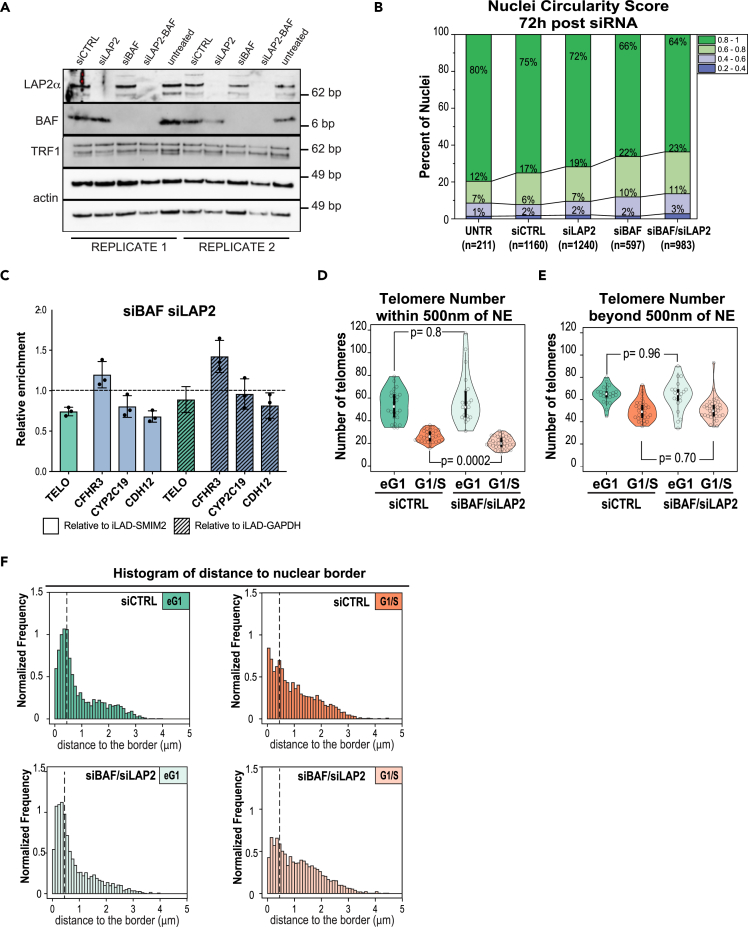
Depletion of LAP2α/β and BAF by siRNA affects 3D genome organization (A) Western blot of whole-cell extracts showing the decreased protein levels of LAP2α or BAF in cells treated with siRNA against LAP2 (siLAP2) or BAF (siBAF) or both (siLAP2-BAF) compared to scrambled siRNA (siCTRL) or untreated cells. TRF1 is unaffected, actin serves as loading control. Two independent experiments are shown. (B) Changes in nuclear circularity 72 h post-treatment, with an increase in abnormal shapes (circularity between 0.2 and 0.6) in siRNA-treated cells compared to control. Number of analyzed cells is indicated below from at least 2 independent experiments. (C) Relative telomere, LAD-CFHR3, LAD-CYP2C19, and LAD-CDH12 enrichment calculated over iLAD-SMIM2 or iLAD-GAPDH and control condition (set at 1) in BAF- and LAP2-depleted cells. n = 3. Mean ± SD is shown. (D and E) Effect of double siBAF/siLAP2 on the total number of peripheral (D) and internal (E) telomeres in early G1 and G1/S phases, with a significant decrease of periphal telomeres in double-depleted siBAF/siLAP2 cells in G1/S phase. The p value of the Wilcoxon unpaired test is indicated. (F) Distribution of the distance between each telomere and nuclear border in control (*Top*) and siBAF/siLAP2 treated (*Bottom*) nuclei in early G1 and G1/S phases indicating a decreased frequency of telomeres associated to the nuclear envelope (below dotted bar indicating 500 nm cutoff).

We next decided to evaluate the outcome of disturbing the initial recruitment of LAP2α/β and BAF to telomeres. For this purpose, we combined siRNA directed against both LAP2 isoforms and BAF and assessed the consequences using different readouts. We confirmed the efficiency of the knockdown by western blotting using whole-cell protein extracts (Figure 6A). BAF is known to restrict accessibility of nuclear membranes to the surface of chromatin, and its depletion results in a global decrease of the nuclei circularity, because of lobular nuclear protrusions.^54^ We therefore measured the circularity score for siLAP2, siBAF, and siBAF-LAP2 cells. Each single siRNA impacted nuclear shape, with a cumulative effect of the co-depletion (Figure 6B – circularity <0.6). Then, we evaluated the consequences of LAP2 and BAF co-depletion on chromatin organization with MadID. We followed the same process as for the targeted screen, except that we analyzed the enrichment of the LAD domain LAD-CFHR3 together with two additional well-described LADs, CYP2C19 and CDH12, and used two independent iLADs regions, SMIM2 and GAPDH to calculate relative enrichments. Both BAF and LAP2 single depletions increased the lamin B1 contact frequency of LAD-CFHR3 in the screen (Figure 4D), which was further increased upon the combination knockdown (Figure 6C). In contrast, LAD-CYP2C19 and CDH12 were found less enriched at the NE in the double depletion compared to controls. These results were not a consequence of changes in the methylation status of iLADs after siRNA depletion (Figure S6A). The depletion of LAP2 and BAF decreased telomere-NE attachment, also with an additive effect compared to single knockdowns (Figures 4D and 6C). While these results support the implication of BAF and LAP2 in genome organization, they only provide a snapshot of the contact frequencies of a large population of asynchronous cells. To further address this phenotype in single cells and at different phases of the cell cycle, we applied our 3D-SIM spatial modeling approach (Figure 2). In general, morphometric descriptors such as the number of telomeres, their density, and nuclear sphericity were comparable in siRNA cells compared to controls (Figure S6B) and compared to the initial dataset (Figure S1D). Only the size of early G1 nuclei was reduced in siRNA BAF and LAP2, a phenotype that was recovered in G1/S cells (Figure S6B). Surprisingly, we did not observe a significant drop of the fraction of telomeres localized within 500 nm of the NE in early G1 (Figures 6D, 6E, and S6C). This could be explained by the multiple tethering mechanisms foreseen for human telomeres and supported by the results of our screen and previous work.^23^ In addition, while the LAP2 and BAF depletion reached at least 80% in both single and double siRNA, immunostaining on fixed cells highlighted remains of LAP2α signal in anaphase, which remarkably colocalized with telomeres (Figure S5B). This LAP2α leftover found at chromosome tips of LAP2-depleted cells could maintain a certain level of telomere tethering. We did observe, however, consequences of LAP2 and BAF loss in interphase cells. The proportion of telomeres permanently found in proximity of the NE in interphase was reduced after LAP2 and BAF co-depletion (Figures 6D–6F, G1/S phase), and spatial statistical analysis showed reduced positive spatial interaction with the NE (Figure S6C), supporting the decrease in telomere enrichment seen by MadID. Overall, these experiments demonstrate that BAF and LAP2 proteins are involved in setting 3D genome organization. Their loss affects telomere tethering to the NE and changes the organization of LADs regions.

## Discussion

Genome organization is key to cellular identity, and structural nuclear landmarks need to be faithfully transmitted over cell generations in any given cell lineage. Rebuilding a functional G1 nucleus at the end of mitosis is therefore an essential process that requires the orchestration of numerous pathways, from NE reformation to re-establishment of chromosome territories and nuclear compartments. Here, we demonstrate an interplay between proteins associated with the NE and telomeres that drives their spatial organization over the cell cycle in cancer cells.

### Telomeres and NE reformation

Our data indicate that telomeres are part of these discrete chromatin regions that attract a specific set of proteins associated with the NE in late anaphase, to initiate membrane formation. These interactions could represent the initial contacts between telomeric chromatin and the NE that perpetuate after mitotic exit, until early G1. Indeed, we estimated that ∼80% of telomeres are located less than 250 nm from LAP2α/β anaphase patches at the onset of NE reformation (Figure 5B), and that more than 50% of telomeres are localized within 500 nm of the nuclear border in early G1 cells (Figure 1C), which correspond to the estimated thickness of the nuclear lamina. What drives the initial recruitment of LAP2α on anaphase telomeres is unclear. An earlier study proposed that, after NE breakdown, residual foci of lamin B1 were found throughout mitosis at major nuclear compartments, such as chromatin.^55^ These landmarks could be targeted during NE reassembly to impart spatial memory from one cell cycle to the next. Even though we were not able to detect these lamin B1 remnants in our cellular model, it would be of interest to see how telomeres organize with regards to the NE as they enter mitosis. A similar scenario was recently suggested for the heterochromatin mark H3K9me2, which acts as a 3D architectural mitotic guidepost, by orchestrating spatial repositioning of heterochromatin through mitosis.^56^ This could suggest that the initial recruitment of proteins associated with the NE depends on the presence of specific heterochromatin marks at telomeres, such as H3K9me2/3, which are involved in perinuclear heterochromatin attachment to the nuclear lamina.^39^^,^^57^ While a mix of both heterochromatic and euchromatic marks characterizes the epigenetic status of telomeres,^58^ the presence of H3K9me2/3 has been observed at telomeric repeats in several human cellular models.^40^^,^^59^^,^^60^^,^^61^ Notably, H3K9me3 can recruit heterochromatin protein 1 (HP1),^59^ a major actor in tethering heterochromatin to the nuclear lamina.^57^ It was also demonstrated that H3K9me2 is retained through mitosis to reposition LADs at the nuclear periphery in daughter nuclei. During mitosis, H3K9me2 is hidden by the phosphorylation of H3 serine 10 (H3S10P), which drives the release of these regions from the nuclear periphery.^56^ Genetic tools could be used to assess the role of these marks on telomere tethering, such as the forced enrichment of HP1α at telomeres, already known to induce telomere structure irregularity.^60^

### 3D telomere distribution is not random

Using 3D spatial statistical modeling, we found that telomeres in cancer cells obey a complex spatial organization in the nuclear volume and showed that, beyond a radial organization, they follow a globally repulsive, regular distribution associated in early G1 with mutual attraction in the short-distance range. While repulsion was maintained throughout the cell cycle, local attraction vanished beyond early G1 and the radial patterning dynamically evolved throughout the cell cycle, suggesting independent determinants for these spatial organization features.

The physical location of telomeres at the extremities of the p- and q-arms of each chromosome could potentially account for the spatial repulsion between telomeres, though it can be expected that the corresponding genomic distance would at least partially be compensated by the Rabl-like organization we noticed in early G1. Since chromosomes are organized into distinct, non-overlapping chromosome territories in interphase nuclei,^62^ an alternative is that repulsion would result from the physical separation of telomeres located on distinct chromosomes, in particular if telomeres are non-uniformly distributed relatively to their respective chromosome territories. The localization of telomeres at the periphery of their chromosome territories,^63^ the polar organization of chromosome territories with opposite centromeric and telomeric ends,^21^ and non-random orientations of telomeric ends^64^ are consistent with a non-random positioning of telomeres relatively to their respective territories. The maintenance of long-range repulsion we observed during the whole cell cycle as opposed to the vanishing short-range attraction after early G1 is consistent with previous reports of absence of large-scale reorganization of chromosome territories beyond G1 but of more dynamic patterns at the sub-chromosomal scale.^63^^,^^65^^,^^66^^,^^67^

Thanks to the resolution offered by 3D-SIM and the large number of characterized nuclei, we were able to achieve a high-resolution analysis of the distance between telomeres and nuclear periphery. This revealed a bimodal distribution showing the existence throughout the cell cycle of two sub-populations of telomeres, one peripheral and one more internal. Chromosome territories in interphase nuclei distribute radially according to size, gene density, or transcriptional activity.^68^^,^^69^^,^^70^ Given the non-random localization of telomeres with respect to their territory, concentric layers of chromosomes could thus induce different modes in telomere distance to nuclear border. Our spatial analyses also revealed that this interaction in 3D between telomeres and the nuclear border is dynamic, from a preferentially peripheral location in early eG1 to a preferentially internal one in G2. A recent study describes a similar behavior for a subset of LADs, with interactions between distal LADs and nuclear periphery that decreased during cell cycle.^71^

### Inheritance of chromosome positions through mitosis

While it is well admitted that chromosomes in mammalian cells form territories, i.e., mutually exclusive globular volumes within the nuclear volume, their precise transmission from mother to daughter cells is still controversial.^67^^,^^72^^,^^73^ Earlier work using elegant 4D imaging suggested that chromosome positions are transmitted through mitosis.^74^ However, it was also reported that the position of chromosome domains is not precisely passed on through mitosis but is rather plastic and redefined during early G1.^75^ Since the nuclear lamina serves as an anchor to organize chromatin, studies addressing single-cell genome-lamina interactions based on the DamID technology have been instrumental to shed light on these mechanisms.^38^ Collectively, LADs from all chromosomes cover ∼30%–40% of the mammalian genome,^76^^,^^77^ but only ∼30% of LADs are found at the periphery in each individual cell nucleus.^39^ These results suggest that LAD interactions with the NE are dynamic, with some LADs making permanent contacts, and other being more variable. They also suggest that even if nuclear organization is not entirely conserved and transmitted through cell divisions, a certain level of organization is preserved. Essential events occurring during mitosis, first during chromatin condensation in prophase to expose or hide certain chromatin loci, and then in late anaphase to reform the lamina at accessible discrete sites, surely set 3D organization for the next interphase. Here, we show that telomeres, and probably extended regions at the ends of linear chromosomes, have this property. One interesting hypothesis is that association of telomeres with forming nuclear lamina in telophase could pull on the corresponding chromosome during nuclear expansion in early G1 and favor its peripheral position. In agreement with this view, genomic repositioning assays using ectopic LAD- or non-LAD-derived sequences expressed in cells clearly showed that mitosis is required for the interaction of an LAD sequence and the lamina.^78^ While the ectopic LADs were localized at the periphery of the condensed chromosomal regions in early stages of mitosis, non-LAD sequences were found within the chromatin mass. This organization of condensed chromatin might favor accessibility in later stages, when the lamina is reforming around chromatin. Indeed, at the end of anaphase, a striking colocalization of lamin B1 with the LAD-derived sequence was observed,^78^ similar to what we observe between telomeres and LAP2α.

### Molecular mechanisms driving telomere-NE attachment

The targeted screen we present here revealed factors at play to tether telomeres to the NE. This is coherent with the fact that mechanisms by which peripheral heterochromatin is tethered to the lamina are redundant,^57^^,^^79^^,^^80^ with at least three mechanisms that rely on adaptor complexes that include proteins such as BAF, LAP2α, or lamin B Receptor. Additional experiments should be performed to further characterize the function of the targets we identified in the screen, and to determine whether they are conserved in other cellular models such as primary cells. DamID has again been instrumental to further dig into the redundancy of mechanisms involved in peripheral chromatin positioning. When applied with the methyltransferase fused to lamin B1, lamin B2, lamin A, or BAF, highly similar maps of LADs were obtained. Additional experiments modulating the levels of lamin A and BAF revealed that these tethering proteins compete for LAD binding.^51^ In contrast, we did not observe an increased frequency of contacts between telomeres and LaminB1 after depletion of lamin A or BAF, as if alternative mechanisms could not compensate each other. Whether telomeres from specific chromosome arms, known to carry distinctive subtelomeric regions, are tethered by different pathways remains to be addressed. While centromeres kept their peripheral localization, the enrichment of telomeres at the nuclear rim was largely lost as soon as cells reached interphase, once nuclei regained their mature size and shape. A similar behavior was recently described for LADs using pA-DamID, a modified version of the CUT&TAG method^81^ combined with DamID.^71^ Applied to synchronized cells, the authors could analyze the cell-cycle dynamics of lamina-associated DNA sequences. They proposed that distal chromosome regions up to ∼25 Mb from telomeres are in contact with the nuclear lamina within the first hours after mitosis, before they gradually move away from the nuclear edge.

A small subset of telomeres retained their NE anchoring throughout interphase. Further experiments would be needed to determine whether the same subset of telomeres is retained there or whether they are interchangeable. Using MadID combined with whole-genome sequencing performed on asynchronous cells, we could previously highlight a preferential enrichment of some subtelomeric regions close to lamin B1,^30^ corresponding to middle- or late-replicating telomeres.^82^ It is likely that the mechanisms of telomere tethering differ whether they occur during postmitotic assembly, or throughout interphase. This could explain why the proximity of telomeres to the reforming NE in telophase persisted after LAP2 proteins and BAF depletion, but we still observed a change in telomere distribution in interphase.

### Limitations of the study

The targeted screen using MadID revealed that several factors impacted telomere or LAD anchoring to the NE, but how they do so still remains to be unraveled. In addition, while the screen was quite extensive, unexpected candidates that could play a role in chromatin anchoring to the nuclear envelope might not have been included.

## STAR★Methods

### Key resources table

**Table:** 

REAGENT or RESOURCE	SOURCE	IDENTIFIER
**Antibodies**		

actin	Santa Cruz	Cat# sc-69879, RRID:AB_1119529
Actin-HRP	Santa Cruz	Cat# sc-47778, RRID:AB_626632
BAF	Santa Cruz	Cat# sc-166324, RRID:AB_2061087
Cep152	SIGMA	Cat# HPA039408, RRID:AB_1079528
Chmp2A	Proteintech	Cat# 10477-1-AP, RRID:AB_2079470
Chmp2B	Abcam	ab33174, RRID:AB_2079471
Chmp4B	Santa Cruz	Cat# sc-82556, RRID:AB_2079484
CREST	Immunovision	Cat# HCT-0100, RRID:AB_2744669
Emerin	Santa Cruz	sc-393247
GAPDH	Proteintech	Cat# 60004-1-Ig, RRID:AB_2107436
GFP FluoTag-X4 atto488	NanoTag	Cat# N0304-At488-S, RRID:AB_2744629
Kinesin 3A	Santa Cruz	Cat# sc-135960, RRID:AB_2132055
Kinesin 5B	Abcam	Cat# ab167429, RRID:AB_2715530
Lamin A/C mouse	Santa Cruz	Cat# sc-7292, RRID:AB_627875
Lamin A/C rabbit	Sigma	Cat# L1293, RRID:AB_532254
Lamin B1	Abcam	ab16048, RRID:AB_443298
Lamin B Receptor	Abcam	ab32535, RRID:AB_775968
LAP2a	Sigma	Cat# SAB4200238, RRID:AB_10794064
LAP2b	Bethyl	A304-840A
Man-1	Bethyl	A305-251A-T
N6-methyladenosine	Synaptic Systems	Cat# 202 003, RRID:AB_2279214
Numa	Bethyl	Cat# A301-510A, RRID:AB_999641
NUP153	Santa Cruz	Cat# sc-101544, RRID:AB_2157327
PINX1	Bethyl	A304-389A
Pom121	Sigma	SAB2700248
POT1	Abcam	ab47082, RRID:AB_882112
RAP1	Bethyl	Cat #A300-306A, RRID:AB_162721
SUN1	Sigma	Cat# HPA008346, RRID:AB_1080462
SUN2	Abcam	Cat# ab87036, RRID:AB_1952674
Tankyrase	Santa Cruz	Cat# sc-365897, RRID:AB_10844977
TIN2	Novus	Cat# NB600-1522, RRID:AB_2205096)
TPP1	Abcam	Cat# ab57595, RRID:AB_2222688
TRF1	Home made	N/A
TRF2	Abcam	Cat# ab13579, RRID:AB_300474
V5	Cell Signaling	Cat #13202
Donkey anti-Human IgG (H+L) Cross-Adsorbed Secondary Antibody, DyLight 488	Invitrogen	Cat# SA5-10126, RRID:AB_2556706
Horse Anti-Mouse IgG Antibody (H+L), DyLight 488	Vector Labs	DI-2488-1.5
Donkey anti-Human IgG (H+L) Cross-Adsorbed Secondary Antibody, DyLight 550	Invitrogen	Cat# SA5-10127, RRID:AB_2556707
Donkey anti-Mouse IgG (H+L) Cross-Adsorbed Secondary Antibody, DyLight 550	Invitrogen	Scientific Cat# SA5-10167, RRID:AB_2556747
Horse Anti-Mouse IgG Antibody (H+L), DyLight 549	Vector Labs	DI-2549-1.5
Donkey anti-Rabbit IgG (H+L) Cross-Adsorbed Secondary Antibody, DyLight 550	Invitrogen	Cat# SA5-10039, RRID:AB_2556619
F(ab')2-Goat anti-Rabbit IgG (H+L) Cross-Adsorbed Secondary Antibody, Alexa Fluor 647	Invitrogen	Cat# A-21246, RRID:AB_2535814
Horse Anti-Mouse IgG Antibody (H+L), DyLight 649	Vector Labs	DI-2649-1.5
Rabbit monoclonal anti-Snail	Cell Signaling Technology	Cat#3879S; RRID: AB_2255011
Mouse monoclonal anti-Tubulin (clone DM1A)	Sigma-Aldrich	Cat#T9026; RRID: AB_477593
Rabbit polyclonal anti-BMAL1	This paper	N/A

**Chemicals, peptides, and recombinant proteins**		

16% formaldehyde solution (W/v), methanol-free	Thermo Scientific	28908
D-Sorbitol	Sigma-Aldrich	97336
SlowFade Diamond Antifade Mountant	Invitrogen	S36963
Glycine	Sigma-Aldrich	G8898
Tween	Calbiochem	655205
Triton	Sigma-Aldrich	x-100
Dulbecco’s Phosphate Buffer Saline DPBS 10x	Gibco	14200-067

**Deposited data**		

3D-SIM images that have been generated in this work are publicly available	https://doi.org/10.57745/IQYEQS.	3D-SIM images that have been generated in this work are publicly available
Fiji & CellProfiler tools	https://github.com/DeboraOlivier/Telomere3D/	Fiji & CellProfiler tools
All Fiji/ImageJ (Schindelin et al., 2012)^83^ & CellProfiler 4.0.7 (Stirling et al., 2021)^84^ image analysis tools can be found at	https://github.com/DeboraOlivier/Telomere3D/	All Fiji/ImageJ (Schindelin et al., 2012)^83^ & CellProfiler 4.0.7 (Stirling et al., 2021)^84^ image analysis tools can be found at
Pipelines for the BIP software are available online on Recherche Data Gouv INRAE	https://doi.org/10.57745/0YF4AI	Pipelines for the BIP software are available online on Recherche Data Gouv INRAE

**Experimental models: Cell lines**		

HeLa 1.2.11 cells	ATCC	Subclone of HeLa CCL-2

**Oligonucleotides**		

Telomeres TelG: 3′-ACACTAAGGTTTGGGTTTGGGTTTG GGTTTGGGTTAGTG-5′ TelC 3′-TGTTAGGTATCCCTATCCCTATCCCTA TCCCTATCCCTAACA-5′	Kychygina et al.^40^	N/A
LAD-CFHR3 FW 3′-TTGGAAGAAGAGAAAGACAAGG-5′ REV 3′-GCAGTGGATGTTTCTCAGCA-5′	Kind et al.^39^	N/A
LAD-CYP2C19 FW 3′-GGATGAGCTTTGCAGGAGAT-5′ REV 3′-AAGCTGTGAGCCTGAGCAGT-5′	Kind et al.^39^	N/A
LAD-CDH12 FW 3′-TTTTTCCTCCCAGGTGACAG-5′ REV 3′-TGATAGCACCTGGGTTAGCAC-5′	Kind et al.^39^	N/A
interLAD-SMIM2 FW 3′-GAAGGTTCCCCCACAGAAAT-5′ RV 3′-CTGAGGCAAAGACAGGGAAG-5′	Kind et al.^39^	N/A
interLAD-GAPDH FW 3′-CTGACTTCAACAGCGACACC-5′ RV 3′-CGCCAGACCCTGCACTTTT-5′	This study	N/A
Farnesyltransferase alpha FW 3′-CTGTACAGGGACAGAGCAGAATG-5′ RV 3′-GATCTGGACCACGGGATTGG-5′	This study	N/A
Farnesyltransferase beta FW 3′-GAGCCGCTGTACAGTCTGAG-5′ RV 3′-CTTTTGCCTGTTCTATGGACGTG-5′	This study	N/A
Nesprin 1 FW 3′-AGCTGGGAAAGGTCAACGAC-5’ RV 3′-TTCAGCTTCTTCACCCTGGC-5’	This study	N/A
Nesprin 2 FW 3′-TTCGACGAGGTAGACTCGGG-5’ RV 3′-CACTGCTCTGAACTGCTTTGC-5’	This study	N/A
Nesprin 3 FW 3′-GGCATCGTCGACGCGAA-5’ RV 3′-AGCTCCTGCAGCTTCGATTT-5’	This study	N/A
Elys FW 3′-GAGATGCTGTGACGGACCC-5’ RV 3′-TCGCATACTTCCACTGAACGG-5’	This study	N/A
Kinesin 20B FW 3′-CGGCAAATTAAAGAGAGAAAGATGC-5’ RV 3′-CAGTGAATATGCTGTGACTTCTAC-5’	This study	N/A
Lem2 FW 3′-CCCACCCTTGGCTTGGTAATG-5’ RV 3′-CTTGGCCTGACAGAACTCATCTG-5’	This study	N/A
Actin FW 3′-AGAGCTACGAGCTGCCTGAC-5′ RV 3′-AGCACTGTGTTGGCGTACAG-5′	This study	N/A
TBP FW 3′-TTCGGAGAGTTCTGGGATTG-5′ RV 3′-GAAAATCAGTGCCGTGGTTC-5′	This study	N/A
Beta 2M FW 3′-AAAGATGAGTATGCCTGCCG-5′ RV 3′-CCTCCATGATGCTGCTTACA-5′	This study	N/A
RPL P0 FW 3′-GGCGACCTGGAAGTCCAACT-5′ RV 3′-CCATCAGCACCACAGCCTTC-5′	This study	N/A
Farnesyltransferase alpha FW 3′-CTGTACAGGGACAGAGCAGAATG-5′ RV 3′-GATCTGGACCACGGGATTGG-5′	This study	N/A
See Table S1 for siRNA references used in this study	Dharmacon smartpool	N/A

**Recombinant DNA**		

pRetroX-PTuner DD-linker-M.EcoGII	Sobecki et al.^30^	ADDGENE #122082
pRetroX-PTuner DD-linker-M.EcoGII-v5-Lamin B1	Sobecki et al.^30^	ADDGENE #122083
pRetroX-PTuner DD-M.EcoGII-v5-Telomeric repeat-binding-factor1	Sobecki et al.^30^	ADDGENE #122084

**Software and algorithms**		

Huygens Essential (svi.nl)	21.10	https://svi.nl/Homepage
Fiji/ImageJ	N/A	https://imagej.net/software/fiji/
Fiji/ImageJ macro batch z projection	N/A	https://www.imperial.ac.uk/medicine/facility-for-imaging-by-light-microscopy/software/fiji/
CellProfiler 4.0.7 (Stirling et al., 2021)^84^	4.0.7	www.cellprofiler.org
Origin, OriginLab Corporation	2019	https://www.originlab.com/
BIP Software	Laboratory of P. Andrey	http://free-d.versailles.inra.fr/html/bip.html
BIP Software Pipelines	This paper	https://doi.org/10.57745/0YF4AI
R Software	R Core Team (2020)	https://www.R-project.org/

**Other**		

1.5H coverslips 22 x 22mm	Marienfeld	0107052
1.5H coverslips 18 mm diameter	Marienfeld	0117580
Unfrosted slides Menzel Gläser SUPERFROST ground edges	Thermo Scientific	Lot 5124873
Cargille oil	Cargille Laboratories	20130

### Resource availability

#### Lead contact

Further information and requests for resources and reagents should be directed to and will be fulfilled by the lead contact: Laure Crabbe (laure.crabbe@univ-tlse3.fr).

#### Materials availability

This study did not generate new unique reagents.

#### Data and code availability

• The 3D-SIM images that have been generated in this work are publicly available from https://doi.org/10.57745/IQYEQS.
• Fiji & CellProfiler tools: https://github.com/DeboraOlivier/Telomere3D/. All Fiji/ImageJ^83^ & CellProfiler^84^ 4.0.7 image analysis tools can be found at: https://github.com/DeboraOlivier/Telomere3D/. Pipelines for the BIP software are available online on Recherche Data Gouv INRAE (https://doi.org/10.57745/0YF4AI).
• All other relevant data supporting the key findings of this study are available within the article and its supplemental information files or from the corresponding authors upon reasonable request.

### Experiment model and study participant details

HeLa 1.2.11 cells^23^ are from a subclone isolated from HeLa CCL-2 (ATCC), an immortal human cell line from cervical carcinoma of a 31-year-old female. Cells were grown at 37°C in Glutamax-DMEM (GIBCO) supplemented with 10% (v/v) fetal bovine serum (GIBCO), 1% (v/v) non-essential amino acids (GIBCO) and 1% (v/v) sodium pyruvate (GIBCO), at 7.5% CO_2_ and 5% O_2_. For cell synchronization, cells were treated with 2 mM thymidine (Sigma) for 16h, followed by 3x washes with pre-warmed PBS (Sigma), and released into growth medium for 8h. A second 2mM thymidine treatment was performed for 16h, followed by washes and release as above, and G1/S samples were collected 0h, G2 cells 6h and early G1 cells 8.5h after release. Expression of pRetroX-PTuner vectors carrying M.EcoGII, M-LB1 or M-TRF1 was induced by addition of 1 μM Shield-1 (Aobious) for 24 hours. Protein depletions were carried out by transfection of 5 μM short interfering RNA (siRNA) for 72h using DharmaFECT transfection reagent (Horizon). For co-depletions (3D-SIM samples), the concentrations of siRNA used were: 5 μM siCTRL (“siCTRL”), 2.5 μM siCTRL + 2.5 μM siLAP2 (“siLAP2”), 2.5 μM siCTRL + 2.5 μM siBAF (“siBAF”), 2.5 μM siBAF + 2.5 μM siLAP2 (“siBAF/siLAP2”). Samples were collected at indicated timepoints and processed further (protein extraction, RNA extraction, immunofluorescence).

### Method details

#### MadID

MadID was essentially performed as described.^30^ Briefly, genomic DNA was isolated using the Blood & Cell Culture DNA Midi kit (QIAGEN) according to the manufacturers’ recommendations. Additionally, an RNase treatment was performed for 1 hour at 37°C: 200 μg/mL RNaseA (Sigma), and 2.5 U/mL RNaseA and 100 U/mL RNaseT1 (RNase cocktail, Ambion).

10 μg of genomic DNA were sonicated during 40 cycles (30 s ON/60 s OFF, low intensity) into 200–400 bp fragments using a Bioruptor Plus sonicator (Diagenode). Sonication efficiency was verified by electrophoresis in a 1.5% agarose gel. 3% of sample was taken as an input. After denaturation for 10 min at 95°C and incubation on ice for 10 min, samples were supplemented with 10x m6A-IP buffer (100 mM Na-Phosphate buffer, pH 7.0; 3 M NaCl; 0.5% Triton X-100), 2.5 μg m6A antibody (Synaptic Systems) and rotated overnight at 4°C. Next, 20 μL of protein A Dynabeads (Thermo Fisher Scientific), pre-blocked for 1 hour in 0.5% BSA and 0.1% Tween-20 in PBS, were added and samples rotated at 4°C for 3 hours. Beads were then washed 4 times in 1 mL 1x m6A-IP buffer. Beads and input samples were resuspended in 100 μL digestion buffer (50 mM Tris, pH 8.0; 10 mM EDTA; 0.5% SDS) containing 300 μg/mL proteinase K and incubated for 3 hours at 50°C while shaking. DNA was purified with the QIAquick PCR Purification kit (QIAGEN) and eluted in 50 μL.

#### Quantitative PCR

Samples obtained after MadID were diluted 1:10 before performing qPCR with the primers listed in the key resources table. For LADs and interLADs regions, 3 μL of sample were used in a reaction mix containing 1 μL 10 μM of each primer (0.5 μM final) and 5 μL 2x Light Cycler SYBR Green LightCycler® 480 SYBR Green I Master (Roche). For telomere amplification, the reaction mix was the same except that 0.2 μM of primer TelG and 0.7 μM of primer TelC were used. Amplification cycle for LADs and interLADs regions was: 15 min at 95°C followed by 40 cycle of 15 s at 95°C and 1 min at 60°C. Amplification cycle for telomeres was: 2 min at 50°C followed by 40 cycles of 15 min at 95°C, 15 s at 94°C and 1 min at 54, and an extension step of 30 s at 72°C.

Standard curves with either input or telomeric DNA were included in every qPCR to ensure amplification efficiency close to 100%. Telomeric DNA consisted of 800 bp of telomeric TTAGGG repeats purified from pSP73.Sty11 plasmid, a gift from Titia de Lange (The Rockefeller University, USA), upon EcoRI digestion. Enrichments were calculated using the ΔΔCt method. The first ΔCt corresponds to the difference between the sequence of interest (either a LAD such as LAD-CFHR3 or telomeres) and an iLAD sequence such as iLAD-SMIM2 that is deprived of methylation in M-LB1 cells:

ΔCt=Ct(LADortel)-Ct(iLAD)

The ΔΔCt was then calculated using M.EcoGII samples as a control average, except for Figure 3B were non transduced cells were used as a control:

ΔΔCt=ΔCt(sampleexpressionM.EcoGII-LB1)-ΔCt(sampleexpressingM.EcoGII)

#### RT-qPCR

Messenger RNA (mRNA) extraction and cDNA synthesis were performed using the μMACS One-step cDNA kit (Milteny) according to manufacturers’ recommendations. RT-qPCR reaction mix was performed in 0.5 μM FW primer, 0.5 μM RV primer and 1x Light Cycler SYBR Green LightCycler® 480 SYBR Green I Master (Roche). Amplification cycle was: 10 min at 50°C and 5 min at 95°C followed by 40 cycles of 10 s at 95°C and 30 s at 60°C. Standard curves were performed to verify that amplification efficiency was close to 100%.

#### Western blotting

Whole protein extracts were obtained by lysing cell pellets in 1x laemmli buffer (10% glycerol, 2% SDS, 63 mM Tris-HCl pH 6.8) followed by 2 cycles of sonication of 15 s 30 to 35 μg protein in LDS lysis buffer NuPAGE (Thermo Fisher Scientific) were then heated at 95°C for 10 min and resolved on pre-cast 4–12% SDS-PAGE gradient gels (Invitrogen). Transfer of proteins from SDS-PAGE gels to nitrocellulose membranes was performed by dry transfer using the iBlot™ 2 Gel Transfer Device (Thermo Fisher Scientific) following the manufacturers’ recommendations. Primary antibodies were incubated for 2 hours at RT or overnight at 4°C, followed by 1-hour secondary antibody incubation. Membranes were overlaid with western blotting substrate for 5 min (Clarity™, BioRad) before visualization with a ChemiDoc™ Imaging System (BioRad).

#### m6A immunodot blot

Dot blot of genomic DNA was performed as described^40^ using the BioRad 96-well Bio-Dot® apparatus. Positively charged Amersham Hybond-N+ membranes (GE Healthcare) and Whatman filter papers (GE Healthcare) preincubated with 2x SSC buffer were assembled onto the apparatus. Samples were denatured (98°C for 10 min followed by 10 min incubation on ice) and loaded on the membrane via vacuum blotting, followed by washing of the wells with cold 2x SSC. The membrane was denatured and neutralized sequentially by placing it on top of a Whatman filter paper (DNA face up) saturated with denaturing solution (1.5M NaCl, 0.5M NaOH) for 10 min at RT and neutralization solution (0.5 M Tris-HCl, pH 7.0, 3 M NaCl) for 10 min at RT. The membrane was crosslinked with UV at 120000 μJ/cm^2^ and blocked for 1 hour in 5% non-fat dry milk in 0.1% TBST (0.1% Tween-20 in 1x TBS, pH 7.4). Subsequently, m6A antibody (Synaptic Systems) was diluted to 1:2000 in 5% non-fat dry milk and 0.1% TBST, and incubated overnight at 4°C. Following three washes with 0.1% TBST, an HRP-conjugated secondary antibody was applied for 45 min at RT. Following three washes with 0.1% TBST, the chemiluminescence signal was visualized using the ChemiDoc Imaging System (BioRad). The intensity of the m6A signal was quantified using the ImageJ software.

#### Protein and DNA immunofluorescence

##### For protein immunofluorescence (conventional microscopy)

Cells were plated onto #1.5 coverslips and were fixed at given timepoints in 4% PFA in PBS for 10 minutes followed by permeabilization in 0.5% Triton 100-X in PBS. After three washes of 5 min in 1x PBS, cells were blocked for 30 min in PBG (1x PBS, 0.5% (w/v) BSA, 0.2% (w/v) cold water fish gelatin) followed by incubation with antibodies listed in the key resources table (either overnight at 4°C or 2 hours at RT). After three washes of 5 min in 1x PBS and 45 min incubation with Alexa488/546/647-conjugated secondary antibodies (Invitrogen), coverslips were washed and mounted with Mowiol mounting medium (24% (w/v) glycerol, 9.6% (w/v) Mowiol 4–88, 0.1 M Tris-HCl pH 8.5, 2.5% (w/v) Dabco).

##### For DNA immunofluorescence to visualize m6A *in situ*

After fixation in 4% PFA and permeabilization in 0.5% Triton 100-X, cells were treated with 200 μg/mL RNaseA (Sigma), and 2.5 U/mL RNaseA and 100 U/mL RNaseT1 (RNase cocktail, Ambion) at 37°C for 1 hour. DNA was then denatured (1.5 M NaCl, 0.5 M NaOH) for 30 min at RT and neutralized (0.5 M Tris-HCl, pH 7.0, 3 M NaCl) twice for 5 min at RT. Samples were then washed three times with 1x PBS for 5 minutes at RT before proceeding with blocking and incubation of antibodies as described above.

##### For protein immunofluorescence (3D-SIM imaging, deconvolved widefield & confocal)

Cells were plated onto #1.5 High-Resolution coverslips (Carl Roth) pre-cleaned with a brief 100% ethanol wash and rinsed with distilled H20 prior to cell seeding. Immunofluorescence was performed according to the Kraus et al. protocol^85^ as follows: for fixation, cells were washed 2x with PBS before being transferred in a 6-well plate containing freshly prepared 4% formaldehyde (methanol-free; FA), pre-equilibrated at 37°C, and incubated for 10 min. FA was removed gently from one side, while simultaneously adding PBS + 0.02% Tween (PBSTw) on the other side for step-wise fixative exchange. Samples were washed twice more with PBSTw, quenched for 10min using freshly prepared 20 mM Glycine in H2O, followed by 2x PBSTw washes. After this stage, the samples were either put in PBS and kept at 4°C for further processing at a later time or were permeabilized for 10 min. in 0.5% Triton X-100. Samples were blocked for ∼30 min in PBG before incubation with primary antibody or GFP-nanobody diluted in PBG, face-down on parafilm, in a humidified chamber (see antibody list below). After primary antibody incubation, samples were transferred in a 6-well plate and washed 4x with PBSTw, before being transferred in PBG with secondary antibodies at a dilution of 1:1000 for 45 min to 1h. After 4x PBSTw washes, samples were post-fixed with freshly prepared 4% PFA for 10 min and washed and quenched as described above. Hoechst was used as a DNA-counterstain at concentrations of 1–5 μm/mL for 5 min in H2O. A final wash was performed in H2O prior to mounting to remove any PBS remnant and avoid PBS crystal formation. The list of antibodies used can be found in the key resources table and the combination of primary & secondary antibodies used were: 1:100 GFP-nanobody conjugated with Atto488 (NanoTag) for TRF1-GFP, 1:1000 mouse anti-lamin A/C (Santa Cruz) and horse anti-mouse Dylight549 or donkey anti-mouse Dylight 550, 1:350 rabbit anti-SUN1 (Sigma) and/or rabbit anti-CEP152 (Sigma) and goat anti-rabbit Alexa-647; 1:1000 mouse anti-lamin A/C (Santa Cruz) and horse anti-mouse Dylight488, 1:2000 human anti-CREST (Sigma) and donkey anti-human Dylight550, 1:350 rabbit anti-SUN1 and goat anti-rabbit Alexa-647; 1:100 GFP-nanobody for TRF1-GFP, 1:250 mouse anti-LAP2α and anti-mouse Dylight549, 1:250 rabbit anti-LAP2β and anti-rabbit Alexa-647.

**3D-SIM mounting media**: given that the refractive index of the mounting medium plays a significant role in 3D-SIM image reconstruction quality, we developed a new mounting media based on a clearing agent - Sorbitol - that had been used for 3D-SIM imaging of DAPI-stained cells up to ∼ 20 μm and shown to yield better modulation-to-contrast-to-noise ratio (MCNR).^33^ After empirical screening of additives and secondary antibodies, we used a formulation of 0.8 × 70% wt/wt Sorbitol/PBS + 0.2x SlowFade Diamond (referred to as Sorb70SF) with a measured RI of 1.448 using Mettler Toledo 30PX refractometer at 23°C. Sorb70SF yielded bright and stable signal with DAPI, Dylight488 / GFP / Atto488, Dylight549 and Dylight 650/Alexa Fluor 647 (AF647), which we then used for the subsequent imaging. Sample mounting was performed as follows: coverslips were first pre-mounted in 35% wt/wt Sorbitol/PBS for 10 min, followed by 70% wt/wt Sorbitol/PBS for 5 min on parafilm. Coverslips were gently tapped onto tissue to remove excess sorbitol, mounted on a ∼12–15 μL drop of Sorb70SF placed in the center of ethanol-cleaned unfrosted slides (Mänzel) and left to settle. Excess mountant was removed prior to sealing with nail polish, and the samples were kept at 4°C before imaging. For best results, samples were imaged within 1 week of preparation.

**Cell cycle staging for 3D-SIM samples** was performed using SUN1 speckles and the centrosomal marker CEP152, both stained with AF647 and imaged in the same channel. Early G1 cells were identified by the presence of SUN1 peri-nuclear speckles (Figure S1) and with 2 connected CEP152 rings,^86^ while late S/ early G2 cells had SUN1 signal only at the NE and exhibited 2 clear separated CEP152 rings connected to the NE, indicating the start of centrosome separation.^87^^,^^88^

#### Microscopy

**Widefield imaging** was performed on a OMX-SR (GE Healthcare/Cytiva) equipped with 405-, 488-, 568-, and 640-nm excitation lasers and emitted fluorescence was collected through a 60X 1.4 numerical aperture (NA) oil objective (Olympus) using an immersion oil of 1.514 refractive index (GE Healthcare) onto two pco.edge.4.1 scientific complementary metal-oxide-semiconductor (sCMOS) cameras (PCO), yielding a pixel size of 80 nm laterally. A dichroic was used to separate emitted fluorescence onto two separate light-paths: emitted fluorescence was filtered using 431/31 nm or 528/48 nm emission filters for DAPI and GFP/Atto488/Dylight488 respectively on one camera, and 609/37nm or 685/40nm for Dylight 549 or AF647/Dylight650 on the second camera respectively. Z stacks (z-step of 262 nm) covering the 3D volume of the cells were acquired for multiple positions using the sequential mode “All Z then Channel”.

**3D-SIM images** were also acquired on the OMX-SR described above, with 3D stacks acquired over the whole cell volume with a *z* step of 125 nm and 15 raw images per plane (5 phases, 3 angles). Spherical aberrations were minimized by using immersion oil with refractive index (RI) of 1.516–1.520 for sample acquisitions. Images were acquired in sequential “All Z then Channel” mode: lamin A/C (Dylight549, 568 nm laser), TRF1 (FluoTag-Atto488, 488 nm laser), SUN1 or CEP152 (AF647, 640 nm laser), DNA (Hoechst, 405 nm laser). 3D-SIM raw image quality was assessed using SIMCheck^34^ and the modulation contrast value (MCNR) measured to make sure it was at the right threshold, i.e., >5. We measure a median value of 4.7 for DAPI-405, 13 for TRF1-488, 10 for lamin A/C-550, and 8.9 for SUN1-647.

**Confocal images** for Figures 4 and S6 were acquired on a Leica TCS SP8 equipped with a 63x 1.4 NA oil objective, a pixel size of 58.5 nm and z steps of 298 nm. For Figures S4A and S4B, images were acquired on a ZEISS Airyscan confocal microscope using a 63× oil objective with 1.4 NA and a pixel size of 155.8 nm. For Figure S4F, images were acquired on a ZEISS Airyscan confocal microscope using a 40× oil objective with 1.4 NA and a pixel size of 131.8 nm and z steps of 1μm.

#### Image processing and analysis

**Image Deconvolution** of widefield (Figure 5B) and confocal images (Figures 4 and S7) was performed using Huygens Essential (SVI). For widefield images, the following parameters were used: 50 max iterations, 40 Signal to Noise Ratio (SNR), 0.01 quality criterion and Classic Maximum Likelihood Estimation (CMLE) deconvolution mode. Chromatic shift correction was calculated in Huygens using a control cell stained only with Hoechst / DAPI and imaged sequentially with all lasers and fluorescing in all channels and deconvolved as above. The calculated chromatic shift correction was added to all deconvolved images. For confocal images deconvolution, the parameters were: 10 max iterations, 10 SNR, 0.05 quality criterion and CMLE deconvolution mode.

##### 3D-SIM image processing and quantitative analysis

3D-SIM images reconstruction from raw data was performed with SoftWoRx v6.5.2 (GE Healthcare) using matched optical transfer functions (OTF)^89^ recorded with 1.516 or 1.518 oil depending on sample thickness and Wiener filter settings set automatically, and channel-aligned using the SoftWorRx tool. Quality control on 3D-SIM images was done using different Fiji/ImageJ macros: first, images were thresholded in 16bit using a function within the SIMCheck plugin (^34^, “*1_SIMCheck_THR_DKO.ijm*”) then individual nuclei were cropped to ensure a single nucleus per image (“*2_CropNucleiBoundingBox.ijm*”), and finally the reconstruction quality was assessed via modulation contrast-to-noise ratio (MCNR) of SIMCheck, using a macro kindly provided by Lothar Schermelleh (“*3_SIMCheck_QC_EzMiron.ijm*”), both visually using the MCNR map on the image, and using the average value per image and channel. Only channels with an MCNR value > 6 for the features of interest (lamin A/C, lamin B1, TRF1) were included in the analysis, and were plotted in Figure S1 using “PlotsOfData” (^90^, 2019), available at https://huygens.science.uva.nl/PlotsOfData/). The different channels of acquired 3D-SIM images were split under Fiji and stored in distinct TIFF files for further processing (“*SPLIT-Channels-into-Folders.ijm*”). All subsequent automated image processing procedures were implemented as batched pipelines using the bip software (http://free-d.versailles.inra.fr/html/bip.html).

The lamin A/C-Dylight549 channel was used to determine nuclear boundaries (BIP Pipeline *nucleus.pipeline*). Images were first resampled using linear interpolation to isotropic voxel size of 100 × 100 × 100 nm. Following Gaussian smoothing, a directional closing filter was applied to fill small gaps in the lamin A/C signal. Non-significant minima were removed by computing extended minima followed by minima imposition.^91^ The watershed transform was then applied on the resulting image.^92^ Labeled regions touching the image borders were removed and the nucleus mask was obtained by selecting the largest remaining region. In a few cases, some artifacts remained (small holes or protrusions at the periphery), which were manually corrected under Fiji.

The TRF1-EGFP channel was used to extract telomeres (BIP Pipeline *telomeres.pipeline*). Following Gaussian smoothing, potentially remaining 3D-SIM-echo artifacts were removed using a specifically designed filter. With this operator, voxel values that were below some proportion of the maximal value of their neighborhood were set to 0. An h-maxima operator was applied to remove non-significant intensity peaks. Automatic thresholding was then performed using an in-house operator specifically developed to extract constellations of objects of similar sizes, by selecting the threshold that minimized the coefficient of variation of object size. Following component labeling, telomeres not contained within the nucleus were removed by masking with the binary nuclear mask. The obtained image was used as a mask for the geodesic reconstruction of the unmasked image of labeled telomeres. This ensured that telomeres located close to the boundary of the nucleus were not truncated. In some cases, a few false positives remained in the images and were removed manually.

The CREST-Dylight550 channel was used to extract centromeres (BIP Pipeline *centromeres.pipeline*). Following Gaussian smoothing, a morphological size opening was used to remove small fluorescent spots. A first thresholding at a low threshold was applied to binarize the centromeric domains. A second thresholding at a high threshold was applied in parallel, followed by component labeling. The obtained labeled were then dilated within the mask obtained with the low threshold. This procedure ensured that centromeres in close vicinity were not aggregated under the same label. Masking and geodesic reconstruction were applied as for telomeres to select centromeres located within the nucleus of interest.

Geometrical and morphological descriptors including volumes, center positions (per-object averages of voxel positions), and shape parameters (elongation, sphericity), were extracted from the segmented images. The nuclear boundary was represented as a triangular mesh extracted from the binary nuclear mask with the Marching Cubes algorithm.^93^ This surface was used for computing distances between object centers and nuclear border. Three-dimensional views of nuclear boundary and telomeric or centromeric positions were generated using the 3D viewer of the Free-D software (^94^; http://free-d.versailles.inra.fr).

### Quantification and statistical analysis

#### Statistical spatial analysis

Telomeric and centromeric patterns were compared to distributions expected under theoretical models for the organizations of points (for telomeres) or real-sized objects (for centromeres) according to the methodology we recently developed.^28^ Computer simulations were used to obtain these distributions. Nuclear boundary (triangular meshes) and telomere positions (geometrical centers) were provided as input in these analyses. For centromeres, the measured individual centromere sizes were provided as additional input parameters and used to ensure that the simulated centromere positions were not leading to intersections between centromeres or with the nuclear envelope. Hence, the models took into account all the variables (nuclear shape and size, number and sizes of analyzed objects) that would otherwise bias the spatial analysis. In the completely random model, points or objects were distributed uniformly and independently (up to object intersections) within the nuclear space.^28^ In the orbital model, the relative positioning to the nuclear border was identical between observed and model-predicted patterns. The orbital model was indeed derived from the completely random model by keeping to its observed value the distance between each telomere and the nuclear border.^28^ The measured distances between telomeres and their closest points at the boundary of the nucleus were thus additional parameters provided as inputs to the orbital model. Comparisons between observed distributions of SDIs and expected uniform distributions under a spatial model (completely random or orbital) were performed using the Kolmogorov-Smirnov test of distribution uniformity within the R software^95^ (R Core Team, 2020). All statistical tests were performed at the 5% significance level.

#### Polarity analysis

The three principal axes of each nucleus were computed from its binary mask and the coordinates of each telomere or centromere were expressed in the corresponding coordinate frame. The number N_i_^+^ (N_i_^−^) of telomeres or centromeres with positive (negative) *i*th coordinate was determined. The polarity index along the *i*th axis was computed as max (N_i_^+^,N_i_^−^)/(N_i_^+^+N_i_^−^).

**Nuclear Circularity analysis** was carried out using ImageJ/Fiji^83^ and Cell Profiler,^84^ tools and pipelines can be found at: https://github.com/DeboraOlivier/Telomere3D/. Briefly, widefield images were projected along the z axis according to the maximum intensity using a custom macro from the FILM facility, Imperial College London (https://www.imperial.ac.uk/medicine/facility-for-imaging-by-light-microscopy/software/fiji/). Nuclei and telomeres were segmented using a custom-made CellProfiler pipeline, and the circularity score was calculated for each nucleus after different treatments (untreated, siCTRL, siLAP2, siLAP2 + siBAF) using the *FormFactor* measurement in CellProfiler corresponding to 4∗π∗Area/Perimeter^2^ (Figure 5B), similar to^96^ to quantify nuclear envelope reformation defects upon LEM2 siRNA. The results were then analyzed using Origin2019.

##### Confocal image analysis

Deconvolved confocal images from Figure 4 where analyzed using BIP tool to segment LAP2α and LAP2β regions and telomeres and measure distances to LAP2α/β regions. An Euclidean distance map was computed on the inverted binary mask of LAP2α/β regions, providing the distance between any nucleus position and the closest LAP2α/β-labeled position. This map was considered as an intensity image and intensity measurements were performed over segmented telomeres. For each telomere, the median distance value was retained as a measure of the distance between its center and the edge of the closest LAP2α/βdomain. The closest distance to LAP2 (α or β) was obtained by taking the minimum of the two distances to LAP2α and to LAP2β.

## Supplemental information

Document S1. Figures S1–S6

Table S1. List of siRNA used in this study, related to Figure 4
