# Supplementary material for: Non-random spatial organization of telomeres varies during the cell cycle and requires LAP2 and BAF
Source: iScience. 2024 Feb 28;27(4):109343. doi: 10.1016/j.isci.2024.109343 (PMC10951912; doi:10.1016/j.isci.2024.109343)
Supplement: Document S1. Figures S1–S6 [file mmc1.pdf]

iScience, Volume *27*

Supplemental information

Non-random spatial organization of telomeres

varies during the cell cycle

and requires LAP2 and BAF

Debora Keller, Sonia Stinus, David Umlauf, Edith Gourbeyre, Eric Biot, Nicolas Olivier, Pierre Mahou, Emmanuel Beaurepaire, Philippe Andrey, and Laure Crabbe

FIGURE S1

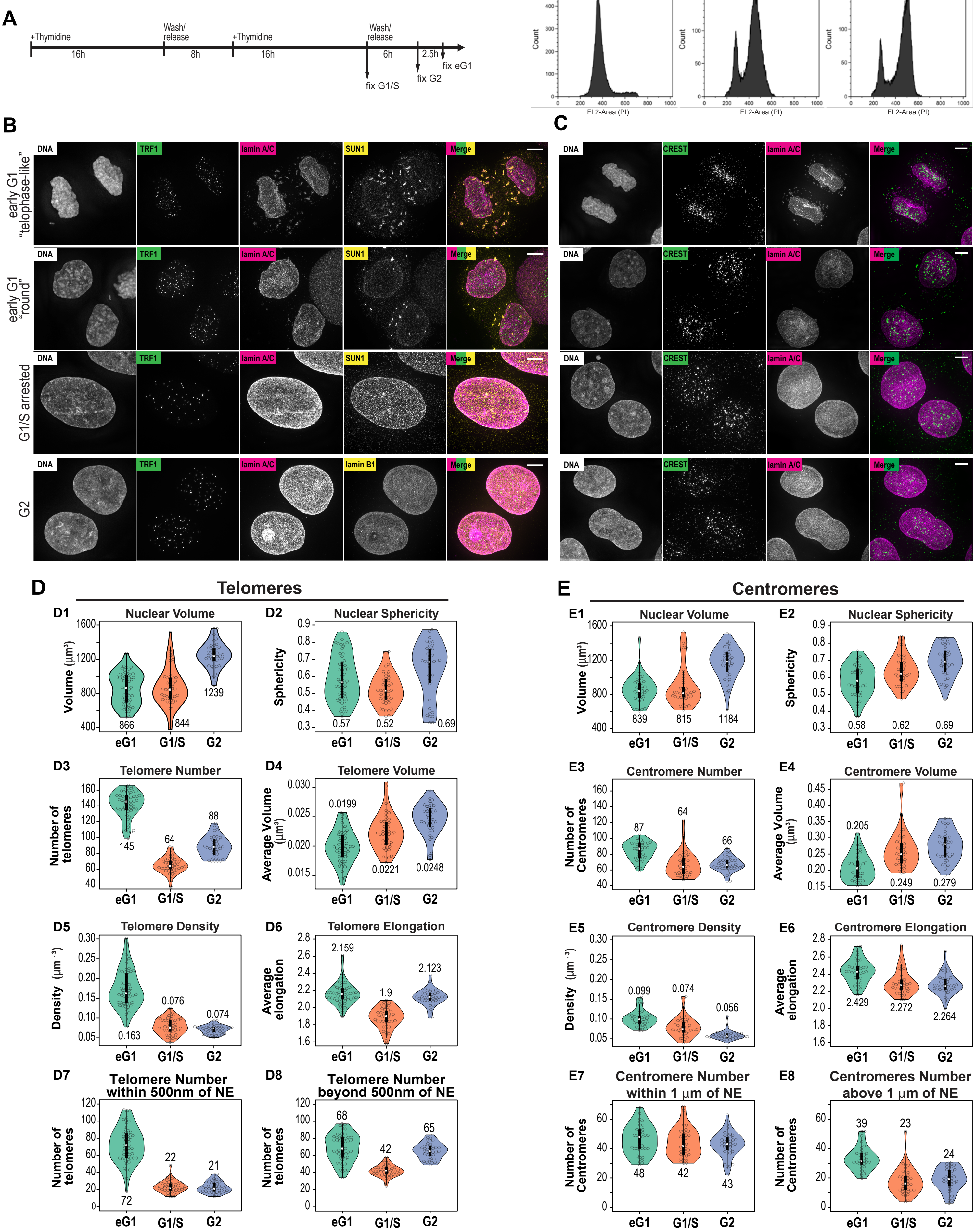
A
+Thymidine
16h
Wash/
release
8h
+Thymidine
16h
Wash/
release
6h
2.5h
fix G1/S
fix G2
fix eG1
G1/S
6h (G2)
8.5h (eG1)
Count
FL2-Area (PI)
B
early G1
"telophase-like"
early G1
"round"
G1/S arrested
G2
DNA
TRF1
lamin A/C
SUN1
lamin B1
Merge
C
DNA
CREST
lamin A/C
Merge
D
Telomeres
D1
Nuclear Volume
Volume (µm³)
866
844
1239
D2
Nuclear Sphericity
Sphericity
0.57
0.52
0.69
D3
Telomere Number
Number of telomeres
145
64
88
D4
Telomere Volume
Average Volume (µm³)
0.0199
0.0221
0.0248
D5
Telomere Density
Density (µm⁻³)
0.163
0.076
0.074
D6
Telomere Elongation
Average elongation
2.159
1.9
2.123
D7
Telomere Number
within 500nm of NE
Number of telomeres
72
22
21
D8
Telomere Number
beyond 500nm of NE
Number of telomeres
68
42
65
E
Centromeres
E1
Nuclear Volume
Volume (µm³)
839
815
1184
E2
Nuclear Sphericity
Sphericity
0.58
0.62
0.69
E3
Centromere Number
Number of Centromeres
87
64
66
E4
Centromere Volume
Average Volume (µm³)
0.205
0.249
0.279
E5
Centromere Density
Density (µm⁻³)
0.099
0.074
0.056
E6
Centromere Elongation
Average elongation
2.429
2.272
2.264
E7
Centromere Number
within 1 µm of NE
Number of Centromeres
48
42
43
E8
Centromeres Number
above 1 µm of NE
Number of Centromeres
39
23
24
eG1
G1/S
G2

Fig. S1 – Related to Fig.1. Sample preparation, imaging, and quantification using 3D-SIM.

A. Timeline showing main steps of sample preparation and fixation after cell synchronization using double-thymidine block (see Star Methods): cells were collected at the end of the 2nd thymidine block (at the G1/S boundary); G2 cells were collected 6h after release and early G1 (eG1) cells were collected 8.5h after release. A representative example of the obtained FACS profile is shown.

B. Representative maximum intensity projection images of nuclei markers imaged using 3D-SIM in sample nuclei at different phases of the cell cycle: DNA (*grey*), TRF1 (*green*), LaminA/C (*magenta*), and SUN-1 (*yellow*) except for G2 stage where LaminB1 (*yellow*) is used. SUN-1 staining is used to distinguish cells in early G1 specifically, which exhibit different morphologies: one "telophase like" with a more compact nucleus, and a "round" one. Merge panel contains LaminA/C (*magenta*), TRF1 (*green*) and SUN-1 (*yellow)* channels. Scale bar: 5 µm.

C. Same as B for centromere 3D-SIM imaging except the following nuclear markers are shown: DNA (*grey*), CREST (*green*) and LaminA/C (*magenta*). Merge panel contains LaminA/C (*magenta*), CREST (*green*) channels. Scale bar: 5 µm.

D. Quantitative 3D image analysis: nucleus size and shape, total number and density of telomeres, number of peripheral and internal telomeres, telomere size and shape during cell cycle. Median values are indicated on graph. (N = 54, 43 and 39 for early G1, G1/S and G2 phases, respectively).

E. Same as D for nuclei with labeled centromeres. (N = 35, 36 and 38 for early G1, G1/S and G2 phases, respectively)

FIGURE S2

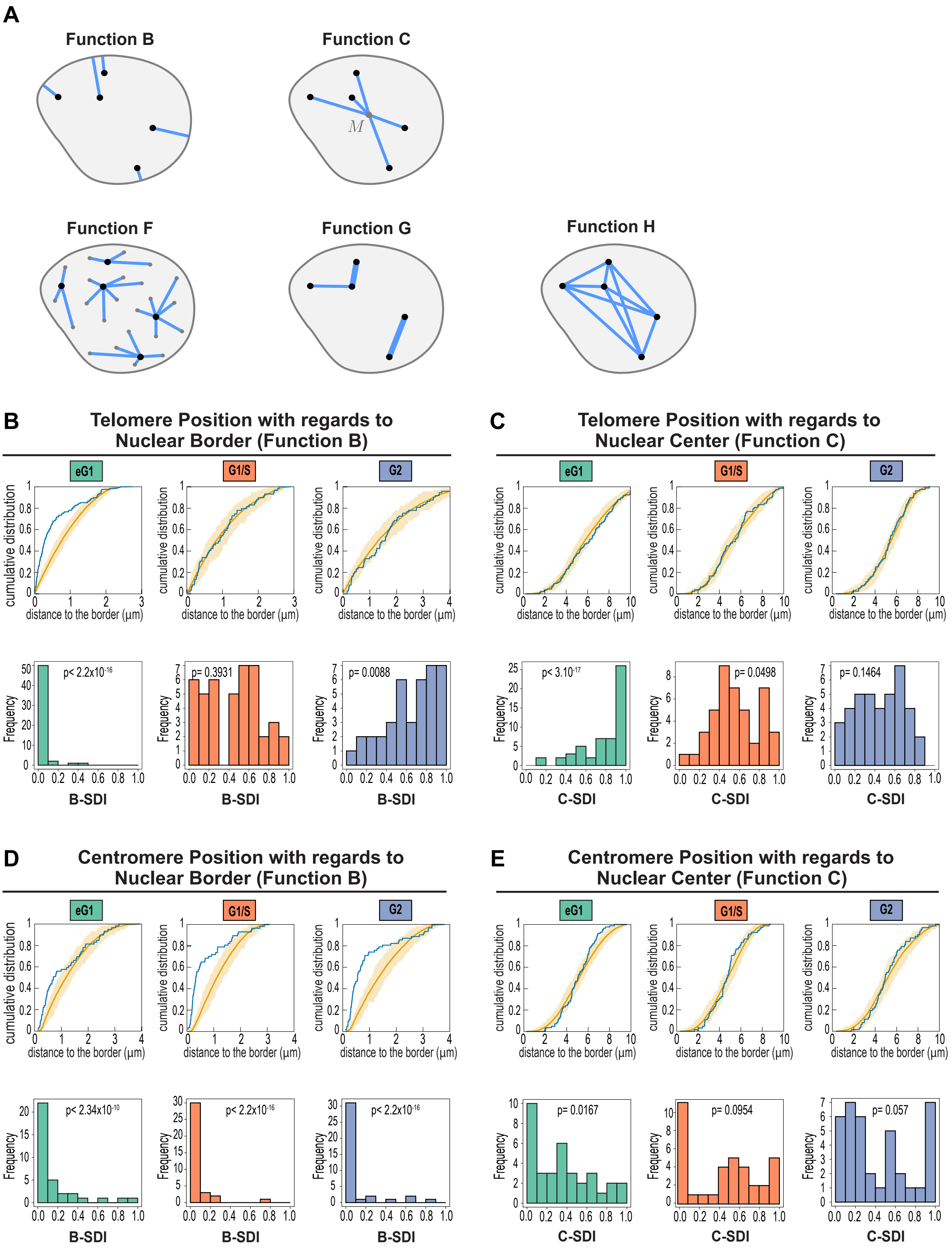

A
Function B
Function C
M
Function F
Function G
Function H
B
Telomere Position with regards to
Nuclear Border (Function B)
C
Telomere Position with regards to
Nuclear Center (Function C)
eG1
G1/S
G2
cumulative distribution
distance to the border (µm)
Frequency
p< 2.2x10-16
p= 0.3931
p= 0.0088
B-SDI
p< 3.10-17
p= 0.0498
p= 0.1464
C-SDI
D
Centromere Position with regards to
Nuclear Border (Function B)
E
Centromere Position with regards to
Nuclear Center (Function C)
p< 2.34x10-10
p< 2.2x10-16
p< 2.2x10-16
p= 0.0167
p= 0.0954
p= 0.057

Fig. S2– Related to Fig.2. Statistical spatial analysis of peripheral and polar organization of telomeres and centromeres during cell cycle.

A. Distance measurements used in spatial statistical analyses. Each telomere or centromere pattern (observed or simulated according to the random or the orbital model) was quantitatively characterized using distribution functions of distance measurements (blue lines). The measurements were: distances to nuclear border (function B); distances from nuclear center (function C); distances between arbitrary positions within the nuclear space and their nearest object (function F); distances to nearest neighbor (function G); distances to all other objects (function H).

B. Individual (*Top*) and population (*Bottom*) analysis of spatial interaction between telomeres and nuclear border. *Top*: observed cumulative distribution function (CDF) of the distance between each telomere and the nuclear border (function B; *Blue curve*) in a representative (median) nucleus at different phases of the cell cycle. *Orange*: average CDF computed over 99 patterns simulated according to a completely random model of telomere distribution. The 95% confidence envelope around the average was computed using another 99 simulated random patterns. *Bottom*: population histograms of the SDI computed using function B and the associated computed p-value (Kolmogorov-Smirnov test of uniformity).

C. Same as B for the analysis of the spatial interaction between telomeres and the center of the nucleus. This interaction was analysed based on the CDF of the distance between each telomere and the nuclear centroid (function C).

D. Same as B for centromeres.

E. Same as C for centromeres.

FIGURE S3

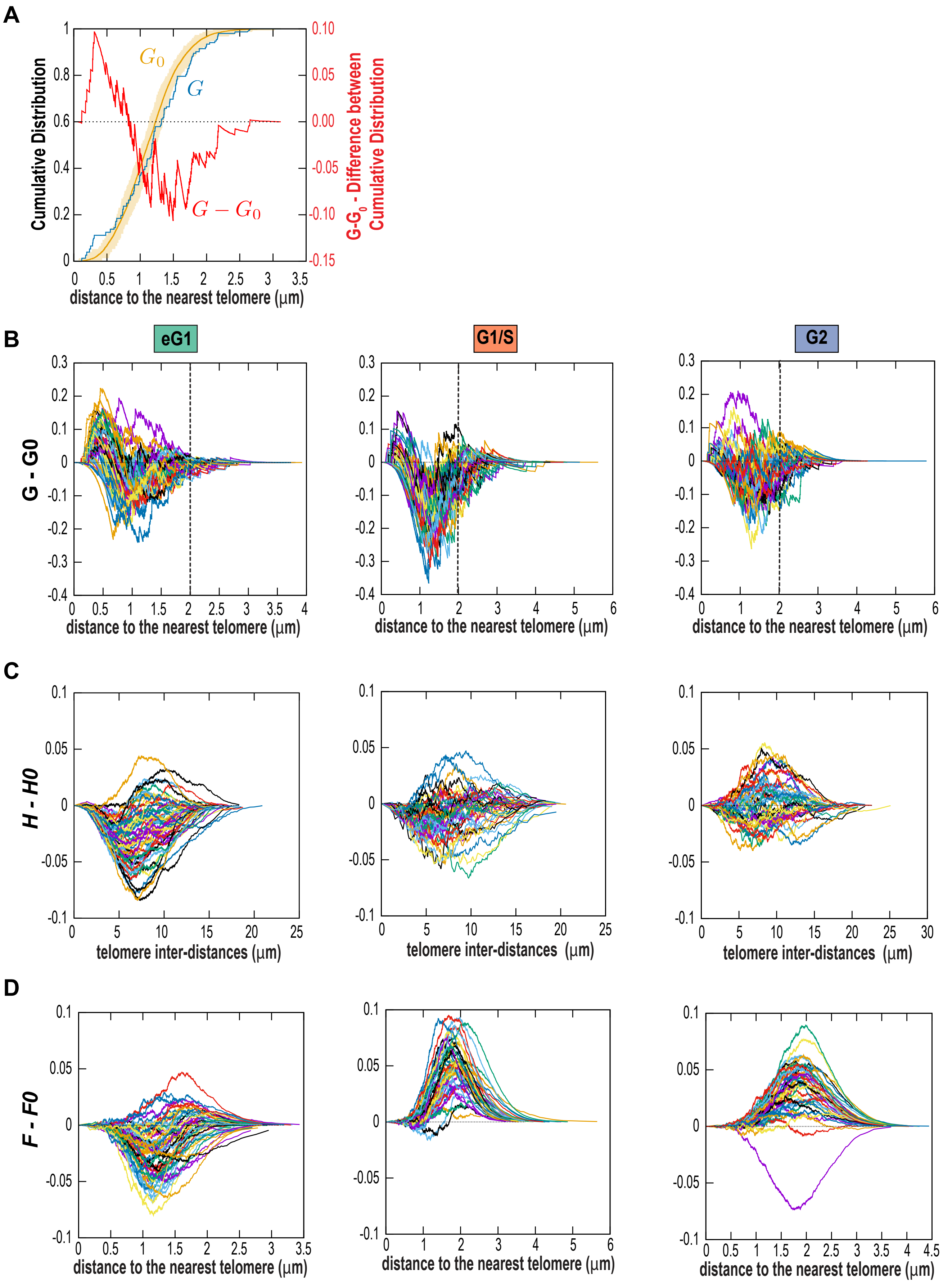

A
Cumulative Distribution
G_0
G
$G - G_0$
G-G0 - Difference between Cumulative Distribution
distance to the nearest telomere (µm)
B
eG1
G1/S
G2
G - G0
distance to the nearest telomere (µm)
C
H - H0
telomere inter-distances (µm)
D
F - F0
distance to the nearest telomere (µm)

Fig. S3. Related to Fig.2. Statistical spatial analysis of peripheral organization of telomeres during cell cycle: difference between observed and predicted distribution functions.

A. Example of difference between the observed function G (Blue) in an individual nucleus at early G1 and the corresponding function G_0 (Orange) according to a completely random model of telomere distribution. The red curve shows the difference between G and G_0.

B. Differences between observed individual functions G and associated model-predicted average functions G. Each color corresponds to a different nucleus. Predictions were computed using a completely random model of telomere distribution. The intervals were G-G0>0 (G-G0<0) highlight an excess (deficit) of measured distances in observed patterns as compared with model predictions. For example, G-G0>0 in the short distance range points to an attractive trend.

C. and D. Same as (B) with functions H and F.

FIGURE S4

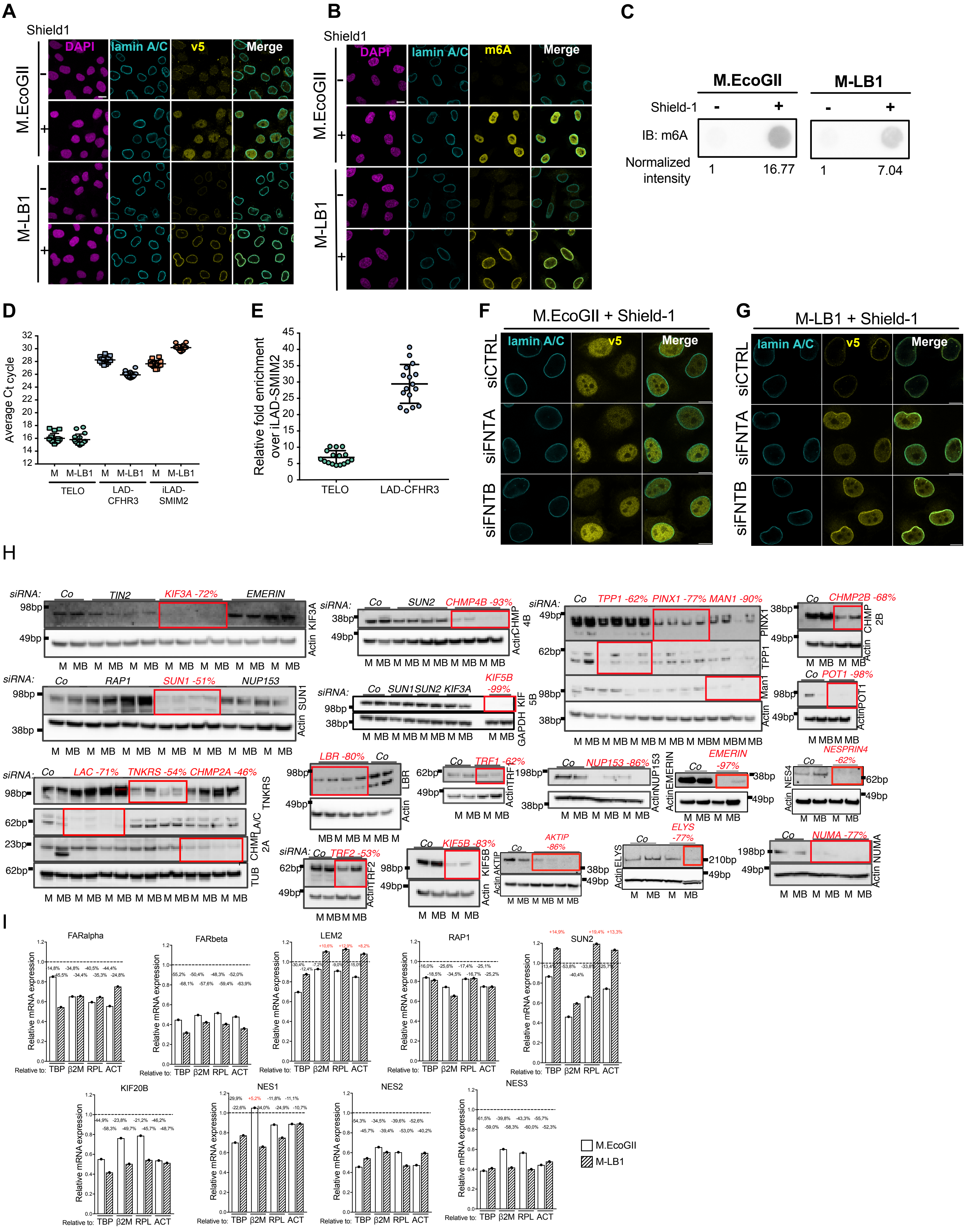
A
Shield1
DAPI
lamin A/C
v5
Merge
M.EcoGII
M-LB1
B
m6A
C
M.EcoGII
M-LB1
Shield-1
IB: m6A
Normalized intensity
1
16.77
7.04
D
Average Ct cycle
TELO
LAD-CFHR3
iLAD-SMIM2
E
Relative fold enrichment over iLAD-SMIM2
F
M.EcoGII + Shield-1
siCTRL
siFNTA
siFNTB
G
M-LB1 + Shield-1
H
siRNA:
I
FARalpha
FARbeta
LEM2
RAP1
SUN2
KIF20B
NES1
NES2
NES3
Relative mRNA expression
Relative to: TBP β2M RPL ACT
M.EcoGII
M-LB1

Fig. S4– Related to Fig.4. M.EcoGII-driven DNA methylation is targeted and detected specifically.

A. Immunofluorescence staining LaminA/C (cyan) and v5-tagged M.EcoGII (v5, yellow) in v5-M.EcoGII or v5-M-LB1 expressing cells, in absence or presence of Shield1 (1 µM, 24h). DAPI is shown in magenta. The middle plane is shown. Scale bar: 10 µm.

B. DNA and protein immunofluorescence staining methylated N6-adenosine (m6A, yellow) and LaminA/C (cyan) in M.EcoGII or M-LB1 expressing cells, in absence or presence of Shield1 (1 µM, 24h). DAPI is shown in magenta. Scale bar: 10 µm.

C. Immunoblot to detect methylated N6-adenosine (m6A) in genomic DNA extracted from M.EcoGII or M-LB1 expressing cells, in absence or presence of Shield1 (1 µM, 24h).

D. Mean Ct values of three technical replicates obtained upon telomere, LAD-CFHR3 or iLAD-SMIM2 amplification by qPCR in control condition. n=16. Mean ± SD is shown.

E. Relative telomere and LAD-CFHR3 enrichment calculated over iLAD-SMIM2 in control condition. n=16. Mean ± SD is shown.

F. Immunofluorescence staining LaminA/C (cyan) and v5-tagged M.EcoGII (v5, yeallow) in control, FARα- or FARβ-depleted M.EcoGII treated with 1 µM Shield1 for 24h. DAPI is shown in magenta. Scale bar: 10 µm.

G. Same as F for M-LB1 expressing cells

H. Western blots of total protein extract from M.EcoGII (M) or M.EcoGII-LaminB1 (MB) expressing cells transfected with the indicated siRNA. Actin was used as a loading control. The estimated percentage of depletion is indicated.

I. Relative mRNA expression of the indicated targets from M.EcoGII (M) or M.EcoGII-LaminB1 (MB) expressing cells transfected with the indicated siRNA. Relative mRNA expression was calculated using different references: Tata Binding Protein (TBP), beta

2 microglobulin (β2M), Ribosomal Protein L (RPL), or Actine (ACT). For simplification, Nesprin 1-3 were labeled Nes 1-3.

FIGURE S5

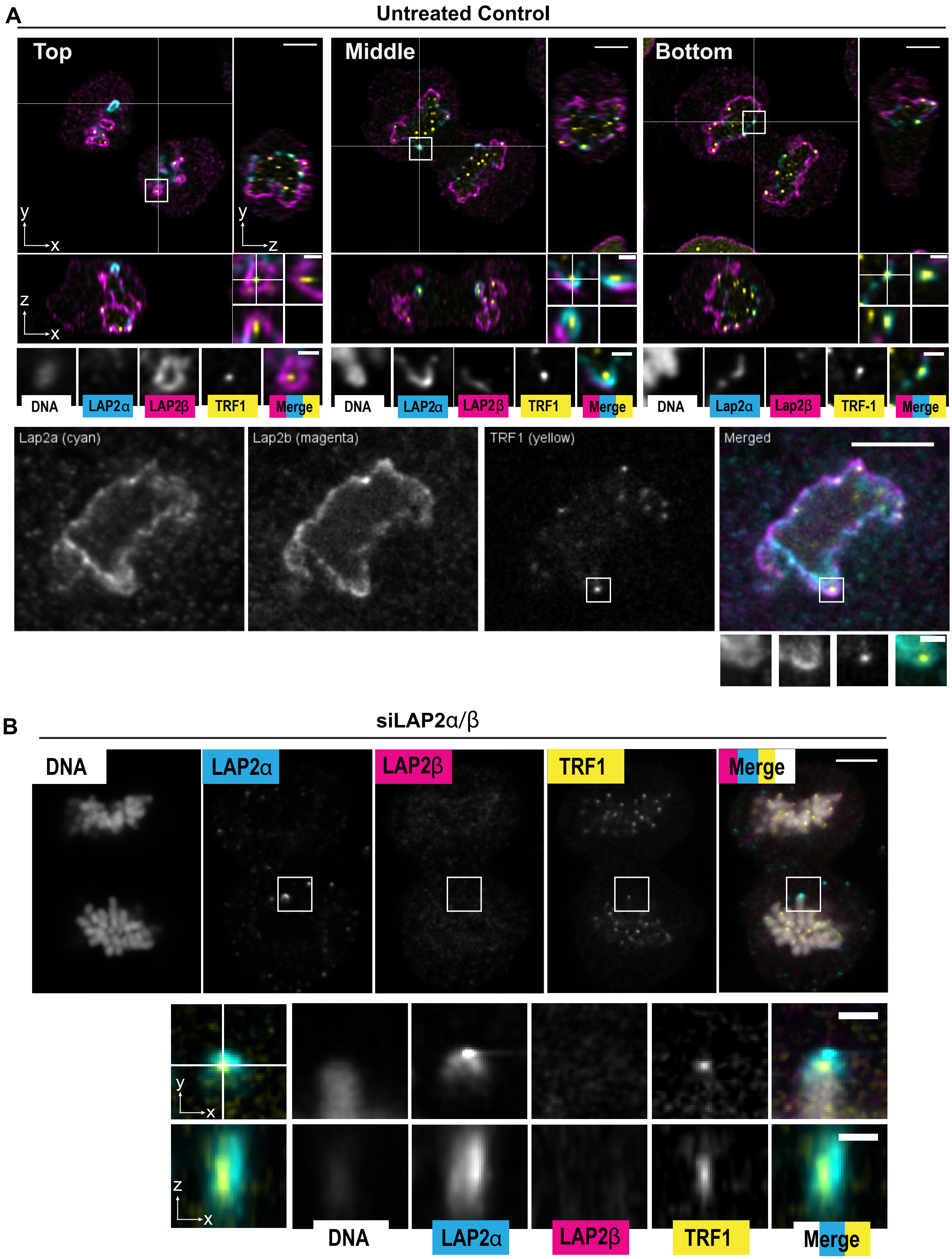
A
Untreated Control
Top
Middle
Bottom
y
x
z
DNA
LAP2α
LAP2β
TRF1
Merge
Lap2α
Lap2β
TRF-1
Lap2a (cyan)
Lap2b (magenta)
TRF1 (yellow)
Merged
B
siLAP2α/β

Fig. S5- Related to Fig. 5. Recruitment of Lap2α and Lap2β at telomeres in anaphase

A. Top panel: orthogonal views of deconvolved confocal images at different cell sections (top, middle, bottom) showing early recruitment of Lap2α (cyan) or Lap2β (*magenta*) proteins preferentially at or around telomeres (*yellow*). DNA in shown in *grey*. Lines indicate planes for orthogonal views in both main and inset.

   Bottom panel: section of a deconvolved anaphase cell, highlighting Lap2α (cyan) recruitment around a telomere (see inset), with Lap2β (*magenta*) exhibiting a slightly different pattern.

B. Decreased signal of Lap2α (cyan) or Lap2β (*magenta*) proteins after siRNA depletion, but remnants of Lap2α are still preferentially recruited around telomeres in early anaphase.

   Scale bars: 5µm in main, 1µm in inset.

FIGURE S6

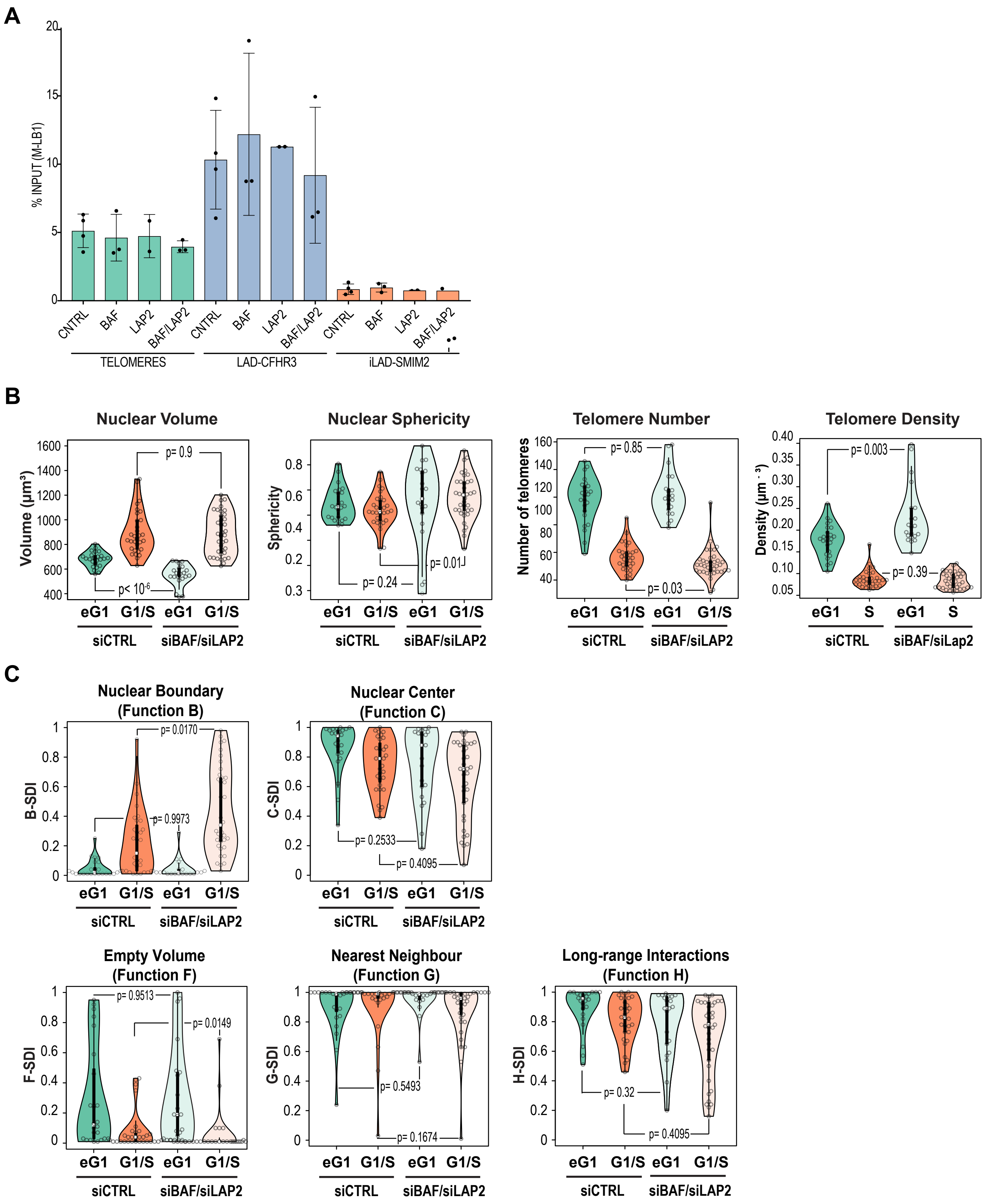

A
% INPUT (M-LB1)
20
15
10
5
0
CNTRL
BAF
LAP2
BAF/LAP2
TELOMERES
LAD-CFHR3
iLAD-SMIM2
B
Nuclear Volume
Volume (µm³)
p= 0.9
p< 10-6
Nuclear Sphericity
Sphericity
p= 0.24
p= 0.01
Telomere Number
Number of telomeres
p= 0.85
p= 0.03
Telomere Density
Density (µm -3)
p= 0.003
p= 0.39
eG1
G1/S
S
siCTRL
siBAF/siLAP2
siBAF/siLap2
C
Nuclear Boundary (Function B)
B-SDI
p= 0.0170
p= 0.9973
Nuclear Center (Function C)
C-SDI
p= 0.2533
p= 0.4095
Empty Volume (Function F)
F-SDI
p= 0.9513
p= 0.0149
Nearest Neighbour (Function G)
G-SDI
p= 0.5493
p= 0.1674
Long-range Interactions (Function H)
H-SDI
p= 0.32
p= 0.4095

Fig. S6-Related to Fig. 6. BAF and LAP2 depletion affect 3D genome organization

A. Telomere, LAD-CFHR3 and iLAD-SMIM2 enrichment in control (n=4), BAF (n=3), LAP2 (n=2) or BAF and LAP2 (n=3) depleted cells. Mean ± SD is shown.

B. Quantitative 3D image analysis of siBAF/siLap2 treated cells in early G1 and G1/S phase (see Methods section): nucleus size and shape , total number and density of telomeres. Statistically significant differences and corresponding p-values are indicated on graph. The comparisons were performed using the Wilcoxon unpaired test.

C. Analysis of spatial interactions between telomeres and nuclear border (*Function B*) or the nuclear center (*Function C*) and between telomeres (*Function F, G, H*) using comparisons to the random model of telomere organization for control or Lap2/BAF siRNA treatments. Function B: distribution SDI computed using the CDF of the distance between each telomere and nuclear border, or *Function C: to the* nuclear center. *Function F-G-H*: same for SDIs computed based on the cumulative distribution function of the distance between each telomere and its closest neighbor (*F*), of the distance between each telomere and any other telomere (*G*), and of the distance between arbitrary nuclear positions and their closest telomeres (*H*). The p-value of the two sample Kolmogorov-Smirnov test is indicated.
